# Optogenetic control of Nodal signaling patterns

**DOI:** 10.1242/dev.204506

**Published:** 2025-05-01

**Authors:** Harold M. McNamara, Alison M. Guyer, Bill Z. Jia, Vicente J. Parot, Caleb D. Dobbs, Alexander F. Schier, Adam E. Cohen, Nathan D. Lord

**Affiliations:** ^1^ Lewis Sigler Institute, Princeton University, Princeton, NJ 08540, USA; ^2^ Department of Computational and Systems Biology, University of Pittsburgh, Pittsburgh, PA 15213, USA; ^3^ Department of Systems Biology, Blavatnik Institute, Harvard Medical School, Boston, MA 02115, USA; ^4^ Department of Chemistry and Chemical Biology, Harvard University, Cambridge, MA 02138, USA; ^5^ Institute for Biological and Medical Engineering, Pontificia Universidad Católica de Chile, Santiago 7820244, Chile; ^6^ Biozentrum, University of Basel, Basel 4056, Switzerland; ^7^ Department of Physics, Harvard University, Cambridge, MA 02138, USA; ^8^ McGowan Institute of Regenerative Medicine, University of Pittsburgh, Pittsburgh, PA 15219, USA

**Keywords:** Nodal signaling, Optogenetics, Zebrafish, Morphogen, Mesendodermal patterning, Gastrulation

## Abstract

A crucial step in early embryogenesis is the establishment of spatial patterns of signaling activity. Tools to perturb morphogen signals with high resolution in space and time can help reveal how embryonic cells decode these signals to make appropriate fate decisions. Here, we present new optogenetic reagents and an experimental pipeline for creating designer Nodal signaling patterns in live zebrafish embryos. Nodal receptors were fused to the light-sensitive heterodimerizing pair Cry2/CIB1N, and the type II receptor was sequestered to the cytosol. The improved optoNodal2 reagents eliminate dark activity and improve response kinetics, without sacrificing dynamic range. We adapted an ultra-widefield microscopy platform for parallel light patterning in up to 36 embryos, and demonstrated precise spatial control over Nodal signaling activity and downstream gene expression. Patterned Nodal activation drove precisely controlled internalization of endodermal precursors. Furthermore, we used patterned illumination to generate synthetic signaling patterns in Nodal signaling mutants, rescuing several characteristic developmental defects. This study establishes an experimental toolkit for systematic exploration of Nodal signaling patterns in live embryos.

## Teaser

**Summary:** A new experimental pipeline for optogenetic control of Nodal signaling in zebrafish embryos raises the possibility to explore systematically how Nodal signaling patterns guide embryonic development..

## INTRODUCTION

Embryos often transmit instructions to their cells using concentration-dependent signaling cues called morphogens. Spatial patterns of morphogen concentration convey positional information to cells, activating position-appropriate developmental programs (Driever and Nüsslein-Volhard, 1988; Gregor et al., 2007; Petkova et al., 2019; Struhl et al., 1989; Stumpf, 1966; Wolpert, 1969; Zagorski et al., 2017). Precisely how cells extract this information from morphogen distributions remains an unanswered question (Kicheva and Briscoe, 2023; Rogers and Schier, 2011). In the classical model, each cell autonomously measures its local signal concentration and selects the appropriate fate in response (Rogers and Schier, 2011; Stumpf, 1966; Wolpert, 1969). However, it has become clear that cells often go beyond simple concentration sensing and instead respond to more complex features of morphogen patterns. For example, cells can pool information via secreted signals to sense signaling domain size in ‘community effects’ (Gurdon, 1988; Nemashkalo et al., 2017) or modify their decisions based on geometric features of their community structure (Muncie et al., 2020). Morphogen dynamics can also carry information; cells can respond differently depending on exposure timing and duration (Camacho-Aguilar et al., 2024; Dessaud et al., 2007; Gritsman et al., 2000; Hagos and Dougan, 2007; Harfe et al., 2004; Johnson and Toettcher, 2019; Kutejova et al., 2009; Sako et al., 2016; Tucker et al., 2008) or whether signaling accumulates abruptly or slowly (Heemskerk et al., 2019; Sorre et al., 2014). Morphogen responses can also be probabilistic, such that the signaling history of a cell determines its fate only in a statistical sense. Indeed, patterning of the zebrafish endoderm and neural tube are initially noisy, only to be refined by downstream processes (Economou et al., 2022; Tsai et al., 2020; Xiong et al., 2013).

Testing quantitative theories of how morphogens organize development requires the ability to systematically manipulate spatial and temporal patterns of signaling activity. Traditional methods can achieve coarse perturbations. For example, genetic knockouts can remove or expand morphogen domains (Lord et al., 2021; Rogers et al., 2017), and microinjections or transplants can introduce point sources of morphogen cues (Müller et al., 2012; Xu et al., 2014). However, the lack of precise spatial and temporal control makes it difficult to explicitly test patterning models. Ideally, an investigator could design and create arbitrary morphogen signaling patterns – in time and space – to rigorously test specific hypotheses.

Optogenetic tools have emerged as a promising strategy for agile and precise control over developmental gene expression (Beyer et al., 2015; LaBelle et al., 2021; Legnini et al., 2023) and signaling (Bugaj et al., 2016; Johnson and Toettcher, 2018; Rogers and Müller, 2020). In an approach pioneered in receptor tyrosine kinase signaling (Grusch et al., 2014), active signaling complexes are assembled by tagging components with protein domains that dimerize in response to light. By rewiring signaling pathways to respond to light, one can, in effect, convert photons into morphogens. Modern optical techniques, in turn, allow light patterning with sub-millisecond time resolution and subcellular spatial resolution (Bugaj and Lim, 2019; Kumar and Khammash, 2022; Repina et al., 2020). In principle, these tools unlock a level of control over developmental signaling that cannot be achieved with traditional manipulations.

In developmental biology, optogenetic strategies have been applied most extensively to investigate terminal patterning via the Ras/ERK signaling pathway in the early *Drosophila* embryo (Ho et al., 2023; Johnson and Toettcher, 2019; Johnson et al., 2017, 2020). These approaches have now been applied to several morphogen pathways (Bugaj et al., 2013; Humphreys et al., 2020; Singh et al., 2022), as well as to vertebrate embryos (Čapek et al., 2019; Rogers et al., 2020; Sako et al., 2016); however, practical challenges have prevented widespread adoption. First, optogenetic reagents often suffer from limited dynamic range. To mimic developmental signaling patterns with light, an optogenetic reagent must switch from negligible background activity in the dark to light-activated signaling levels approaching peak endogenous responses. Second, common strategies for spatial light control have limited throughput and flexibility. Systematic dissection of morphogen signaling mechanisms requires a means to deliver precise patterns of light to large numbers of live embryos as they grow and change shape.

Nodal is a TGFβ family morphogen that organizes mesendodermal patterning in vertebrate embryos (Chen and Schier, 2001; Conlon et al., 1994; Feldman et al., 1998; Schier, 2003). Nodal ligands exert their effects by assembling complexes of type I and type II cell surface receptors, and an EGF-CFC family co-factor (Gritsman et al., 1999; Reissmann et al., 2001; Schier, 2003; Yeo and Whitman, 2001). Ligand-induced proximity between the receptors leads the constitutively active type II receptor to phosphorylate and activate the type I receptor, which then phosphorylates the transcription factor Smad2 (Attisano and Wrana, 2002). Once active, pSmad2 translocates to the nucleus and, in concert with other transcriptional co-factors, induces the expression of Nodal target genes (Dubrulle et al., 2015; Massagué et al., 2005). In zebrafish, the Nodal ligands Cyclops and Squint are produced at the embryonic margin (Dougan et al., 2003; Erter et al., 1998; Rebagliati et al., 1998; Sampath et al., 1998). Cyclops and Squint dimerize with the ubiquitously expressed Nodal ligand Vg1 prior to secretion to form active heterodimeric ligands (Bisgrove et al., 2017; Montague and Schier, 2017; Pelliccia et al., 2017). Diffusion of these ligands from the margin generates a vegetal-to-animal concentration gradient that instructs germ layer fate selection (Chen and Schier, 2001; Lord et al., 2021; Müller et al., 2012); higher Nodal exposure directs cells to endodermal fates, while lower levels direct cells to mesodermal fates (Chen and Schier, 2001; Dougan et al., 2003; Gritsman et al., 2000; Schier et al., 1997; Thisse et al., 2000; Vincent et al., 2003). Recent work also suggests that the Nodal signaling gradient establishes a gradient of cell motility and adhesiveness that is important for ordered cell internalization at the onset of gastrulation (Carmany-Rampey and Schier, 2001; Pinheiro et al., 2022).

Nodal was the first developmental signal to be made optogenetically tractable in zebrafish through fusion of the type I and type II receptors *acvr1b* and *acvr2b* to the photo-associating light-oxygen-voltage-sensing (LOV) domain of aureochrome1 of the alga *Vaucheria frigida* (Sako et al., 2016; Takahashi et al., 2007). Under blue light illumination, dimerization of the LOV domains brings the receptors together and initiates signaling. While these first-generation ‘optoNodal’ reagents enabled temporal control of Nodal target gene expression, spatial patterning of Nodal signaling with light has not yet been reported. Furthermore, LOV domains often exhibit slow dissociation kinetics (Pudasaini et al., 2015), which may limit the temporal resolution with which signals can be controlled, and may also contribute to problematic dark activity. Achieving biologically relevant spatial patterning places more stringent technical requirements on both optogenetic reagents and optical instrumentation than does temporal patterning.

Here, we report an experimental pipeline for optogenetic patterning of Nodal signaling with improved dynamic range, as well as higher temporal resolution, spatial resolution and throughput. We develop improved optoNodal reagents (hereafter optoNodal2) with enhanced dynamic range by fusing Nodal receptors to the light-sensitive heterodimerizing pair Cry2/CIB1N, and by further sequestering the type II receptor to the cytosol. We use a custom ultra-widefield patterned illumination approach (Farhi et al., 2019) for spatial patterning and live imaging of up to 36 zebrafish embryos in parallel. We demonstrate flexible patterning of Nodal signaling activity and target gene expression in zebrafish embryos. We further demonstrate spatial control over cell internalization movements during gastrulation, and partial rescue of several development defects in Nodal signaling mutants. Our platform lays the foundation to systematically dissect the spatial logic of Nodal signaling and demonstrates a generalizable approach to high-throughput optogenetic control over morphogen signals in the zebrafish embryo.

## RESULTS

### Development of new optoNodal reagents with enhanced kinetics and dynamic range

An ideal optogenetic reagent would evoke strong signaling in response to light and no signaling in the dark. In practice, many photo-associating domains exhibit some affinity in the dark, leading to unwanted background activity. The original, LOV-based, optoNodal reagents were highly active in the light, as they were able to induce robust expression of ‘high-threshold’ Nodal expression targets such as *gsc* and *sox32* (Sako et al., 2016). However, we noticed problematic levels of dark activity even when expressed at low doses of mRNA; wild-type zebrafish embryos injected with LOV optoNodal mRNAs and raised in the dark exhibited measurable Nodal signaling activity, as visualized by pSmad2 immunostaining as well as severe phenotypes at 24 hpf, consistent with hyperactive Nodal signaling (Fig. S1A).

**Fig. 1.**
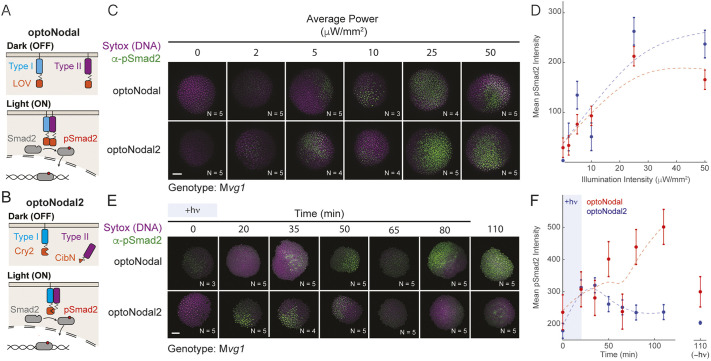
**Improved optoNodal2 reagents based on Cry2-Cib1N heterodimerization.** (A) Schematic of previously developed LOV-based optoNodal reagents (Sako et al., 2016). Type I and type II receptors are tethered to the membrane via a myristoylation motif (top). Blue light induces homodimerization between LOV domains, activating Nodal signaling (bottom). (B) Schematic of OptoNodal2 reagents. The myristoylation motif is removed from the type II receptor, localizing it to the cytoplasm (top). Blue light induces heterodimerization of Cry2 and Cib1N, activating Nodal signaling (bottom). (C) Blue light intensity responses for optoNodal (top row) and optoNodal2 (bottom row) reagents. M*vg1* embryos injected with indicated reagents (15 pg per receptor mRNA) were illuminated for 1 h with 470 nm light at sphere stage at the indicated intensity. Nodal signaling was measured by α-pSmad2 immunostaining (green). Images are maximum intensity projections of representative embryos. Scale bar: 100 µm. Staining heterogeneity likely represents uneven dispersal of injected mRNA. (D) Quantification of Nodal signaling activity from C. α-pSmad2 staining intensity was extracted from segmented nuclei in optoNodal (red) and optoNodal2 (blue) treatment groups; each point represents the average nuclear staining intensity from replicate embryos. Number of replicate embryos for each condition are indicated in the corresponding images in C. Data are mean±s.e.m. Dashed curves depict cubic smoothing spline interpolations. (E) Measurement of response kinetics for optoNodal (top row) and optoNodal2 (bottom row) reagents. M*vg1* embryos injected with indicated reagents (15 pg per receptor mRNA) were illuminated for 20 min with 470 nm light (20 µW/mm^2^ average power) at dome stage. Nodal signaling was measured by α-pSmad2 immunostaining (green). Images are maximum intensity projections of representative embryos. (F) Quantification of Nodal signaling activity from E. α-pSmad2 staining intensity was extracted from segmented nuclei in optoNodal (red) and optoNodal2 (blue) treatment groups; each point represents the average nuclear staining intensity from replicate embryos. Number of replicate embryos for each condition are indicated in the corresponding images in E. Data are mean±s.e.m. Dashed curves depict cubic smoothing spline interpolations. Background intensity of unilluminated embryos at the 110 min timepoint are included (−hν) to indicate baseline levels of signaling activity.

We set out to design improved optoNodal receptors (Fig. 1A,B). Inspired by a recent study on optogenetic TGFβ receptors (Li et al., 2018), we reasoned that dark activity could be reduced by introducing two modifications. First, we replaced the LOV-based photo-associating domains with photo-associating domains from *Arabidopsis* Cry2 and Cib1, which have previously been used to engineer light-driven dimerization events with rapid association (∼seconds) and dissociation (∼minutes) (Kennedy et al., 2010). Second, we removed the myristoylation motif from the constitutive type II receptor so it became cytosolic in the dark. We hypothesized that this change would decrease the effective concentration at the membrane in the dark, reducing the propensity for spurious, light-independent interactions. Indeed, we found that dark activity is greatly reduced over a wide range of mRNA dosages for the redesigned receptors. Embryos injected with up to 30 pg of mRNA encoding each receptor appear phenotypically normal at 24 hpf when grown in the dark (class I phenotypes, Fig. S1C,D). By contrast, embryos injected with optoNodal receptors exhibited high fractions of embryos with axis curvature and loss of head structures (class II phenotypes) or embryo disruption (class III phenotypes).

We next compared the inducibility and kinetics of optoNodal2 relative to previously reported optoNodal. To test the illumination responses, we injected equal amounts of mRNA encoding each set of reagents into mutant embryos lacking endogenous Nodal signaling (M*vg1* mutants) and exposed the embryos to 1 h of blue light illumination with varying intensity using an open-source LED plate (Bugaj and Lim, 2019). Both sets of receptors induced Smad2 phosphorylation over a similar range of powers (saturating near 20 µW/mm^2^, Fig. 1C,D). Notably, the optoNodal2 receptors exhibit equivalent potency without the drawback of detrimental dark activity (Fig. S1A,B). We repeated these measurements at functionally matched mRNA doses – the highest dose of each reagent that was tolerated without gross phenotypes at 24 hpf – and confirmed that optoNodal2 reagents exhibited an improved dynamic range (Fig. S2). To measure dynamic responses, we exposed M*vg1* embryos expressing the two sets of receptors to a 20 min impulse of saturating light intensity (20 µW/mm^2^) and stained for pSmad2 at several timepoints after stimulation (Fig. 1E,F). The optoNodal2 reagents exhibited rapid kinetic responses; pSmad2 levels reached maximal intensity approximately 35 min after stimulation and returned to baseline approximately 50 min later. By contrast, signaling in the optoNodal reagents failed to return to background levels for at least 90 min after cessation of illumination. We confirmed this observation by repeating the dynamic response measurements in an independent mutant background lacking Nodal signaling (Gritsman et al., 1999) (MZ*oep*, Fig. S3). Thus, the optoNodal2 reagents improved the dynamic range and response kinetics over the original optoNodal design without sacrificing potency of light-driven Nodal pathway activation.

### A platform for high-throughput spatial patterning of Nodal signaling activity

Optogenetic tools in developmental biology promise the ability to test spatial and temporal patterns of signaling activity on demand. Recent studies have described spatial modulation of developmental signaling using microscope-coupled digital micromirror devices (DMDs) (Johnson et al., 2020), well as laser scanning over geometrically defined regions of interest (ROIs) (Čapek et al., 2019) and LED illumination with static photomasks (Repina et al., 2020). These approaches have limited throughput and flexibility: most DMD-equipped and laser-scanning microscopes can only address a single embryo at a time, and static photomasks require long turnaround times to design and test new patterns. The ability to flexibly pattern signaling in multiple embryos in parallel would open the possibility of systematically exploring how geometric pattern features guide developmental outcomes.

To achieve this goal, we adapted an ultra-widefield microscope system that has been applied to large-area optogenetic manipulation of mouse brain slices (Farhi et al., 2019) and to study the early electrophysiology of developing zebrafish hearts (Jia et al., 2023) (Fig. S4). The microscope leverages a 4× macro objective lens and DMD projector to address a ∼15 mm^2^ area. The system can project light patterns over eight zebrafish embryos in a single field of view with close to single-cell resolution. We outfitted the microscope with a scanning stage, multi-color LED illuminator and motorized filter wheel to enable simultaneous multi-channel fluorescence imaging and scanning over multiple fields of view. Furthermore, we built a custom microscope interface in MATLAB that enables direct control of each microscope component, paving the way for complex acquisitions that incorporate position scanning, imaging and spatial light patterning. To mount zebrafish embryos for patterning, we 3D-printed embryo mounts that allow blastula and gastrula stage embryos to be arranged in a regular array (Fig. S5). Embryos mounted in this way remain still enough for precise light delivery and they develop normally over 24 h (Movie 1).

**Fig. 2.**
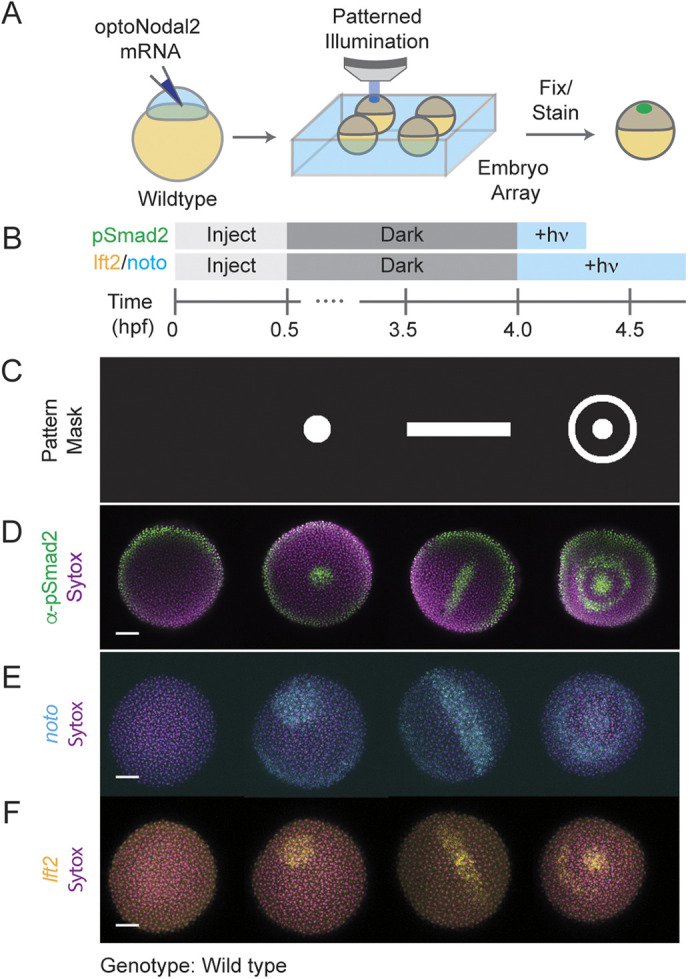
**Platform for spatial and temporal patterning of Nodal signaling activity.** (A) Schematic of patterning experiment. One-cell wild-type embryos were injected with mRNA encoding optoNodal2 receptors (15 pg per receptor). At sphere stage, embryos were mounted in custom array mounts compatible with an upright microscope. Spatial patterns of light were generated using an ultra-widefield microscope incorporating a DMD-based digital projector (Fig. S4). Patterns were applied with average intensity of 20 µW/mm^2^. (B) Experimental timeline. Embryos were injected with optoNodal2 mRNAs (15 pg per receptor mRNA) at the one-cell stage. Embryos were kept in the dark until 4 hpf. Embryos stained for pSmad2 (D) were illuminated from 4-4.3 hpf, while embryos stained for *lft2* or *noto* expression (E,F) were illuminated from 4-4.75 hpf (‘+hν' indicates illumination). All embryos were fixed immediately after light treatment. (C-F) Demonstration of spatial patterning of Nodal signaling activity and target gene expression. (C) DMD pattern masks used for spatial patterning. (D) α-pSmad2 immunostaining (green) demonstrating spatial patterning of signaling activity. (E) Spatial patterning of *noto* gene expression (cyan). (F) Spatial patterning of *lft2* gene expression (yellow). Embryos were double stained for *lft2* and *noto*; each column of images in E and F depict the same embryo imaged in different channels. All images in D-F are maximum intensity projections derived from confocal images of a representative embryo. Scale bars: 100 µm.

To demonstrate the spatial patterning capability of this platform, we projected light patterns – a spot, line or bullseye (Fig. 2A-C) – onto groups of sphere-stage zebrafish embryos mounted in a two-dimensional grid pattern (i.e. with regular spacing between embryos in X and Y dimensions). Precise Nodal signaling patterns, as read out by pSmad2 immunostaining, could be generated for each pattern position with a 20 min stimulation (Fig. 2B,D). Application of patterns for longer times (45 min) induced spatially patterned gene expression of both a gene in the Nodal regulatory pathway [*lefty2* (*lft2*), Fig. 2B,F] and of a Nodal target gene encoding axial mesodermal fate [*flh* (*noto*), Fig. 2E]. The boundaries of *noto* expression patterns were consistently outside of those of *lft2*. We speculate that this effect arises, at least in part, from differential sensitivity of *lft2* and *noto* expression rates to Nodal signaling (Dubrulle et al., 2015), combined with slight light scatter beyond the edges of the nominal projection pattern. Experiments with graded patterns of light (Fig. S6) support this interpretation. Collectively, these results demonstrate that the new optoNodal2 reagents, coupled with an ultra-widefield patterning platform, enable spatial and temporal patterning of Nodal signaling activity and Nodal-dependent gene expression.

### Optogenetic patterning of endodermal cell specification and internalization

**Fig. 3.**
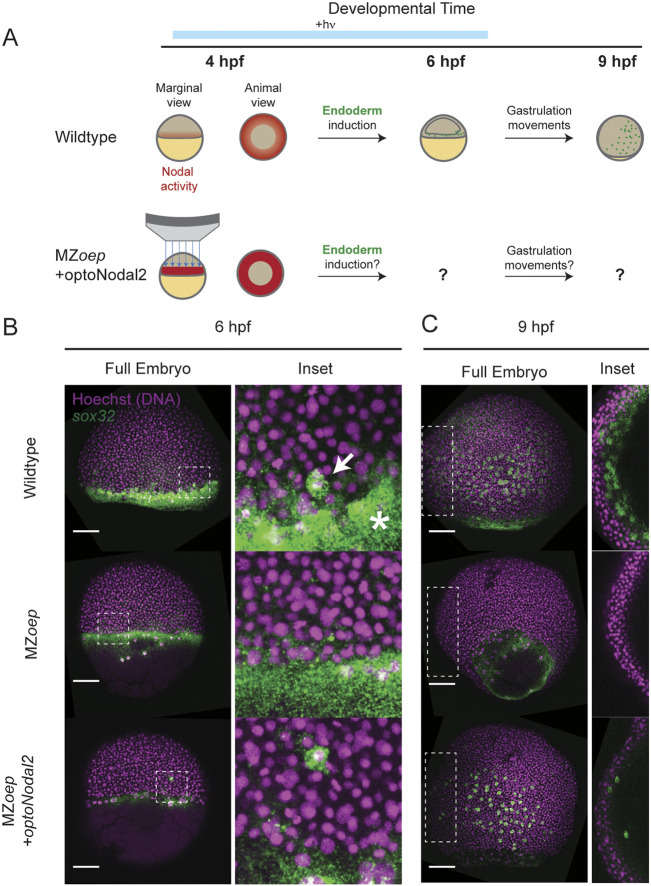
**Rescue of endoderm precursors and internalization movements.** (A) Endoderm rescue experiment. In wild-type embryos, Nodal signaling near the margin turns on the master endoderm transcription factor *sox32* at 4 hpf. By 6 hpf, *sox32*^+^ endodermal precursors have internalized, and by 9 hpf they have spread over the yolk via random walk movements. MZ*eop* mutants lack Nodal signaling and do not specify endoderm. We rescued *sox32* expression and downstream cell movements in MZ*oep* embryos by targeted optoNodal2 stimulation at the margin from 3.75 to 6 hpf (indicated by blue bar). Embryos were injected with 30 pg of optoNodal2 mRNA. Illumination patterns had average powers of 40 µW/mm^2^. (B) Rescue of *sox32* expression at 6 hpf expression with optoNodal2 stimulation*. sox32*^+^ cells were visualized by hybridization chain reaction in wild type (top row), MZ*oep* (middle row) and optoNodal2-stimulated MZ*oep* embryos (bottom row). Insets and white arrow highlight localization of *sox32^+^* cells at the embryonic margin. Asterisk highlights Nodal-independent *sox32* expression in the extra-embryonic yolk syncytial layer. (C) Rescue of cell internalization movements with optoNodal2 stimulation. *sox32*^+^ cells were visualized by HCR at 9 hpf in wild type (top row), MZ*oep* (middle row) and optoNodal2-stimulated MZoep (bottom row). Insets depict maximum intensity projections of middle confocal slices to visualize the hypoblast cell layer. *sox32*^+^ cells reside in the hypoblast in wild-type and optoNodal2-treated embryos at 9 hpf. Scale bars: 100 µm.

We next sought to initiate more-complex developmental programs using patterned Nodal stimulation. In zebrafish, endodermal cells are specified by high levels of Nodal signaling within two cell tiers of the margin, after which they internalize via autonomous ingression at the onset of gastrulation (Carmany-Rampey and Schier, 2001; Liu et al., 2018). We therefore reasoned that optogenetic stimulation targeted to the margin could initiate endodermal specification (i.e. *sox32* expression) and internalization movements in the absence of endogenous Nodal signaling. To test this hypothesis, we injected RNA encoding our optoNodal2 receptors into MZ*oep* mutants and stimulated the margin with targeted illumination from 3.5 hpf (immediately before the onset of Nodal signaling) until 6 hpf (early gastrulation) (Fig. 3A). To visualize specification, internalization and dispersal of endodermal cells, we harvested stimulated and dark-control embryos at 4 hpf, 6 hpf and 9 hpf, and stained for *sox32* mRNA.

Confocal imaging of patterned Mz*oep* embryos revealed a salt-and-pepper pattern of *sox32* induction at the margin at 6 hpf, consistent with its expression pattern in wild-type embryos (compare with Fig. 3B, top and bottom rows). Furthermore, we found that at 9 hpf *sox32*^+^ cells in illuminated Mz*oep* embryos had migrated animally and spread over the yolk, again mimicking the normal distribution of endodermal precursors (Fig. 3C). Importantly, individual confocal sections reveal that the induced *sox32*^+^ cells reside in the hypoblast, consistent with them executing internalization movements at gastrulation (Fig. 3C, right column). In unilluminated MZ*oep* mutants, by contrast, *sox32^+^* cells were absent at all observed stages. Quantification of *sox32^+^* cell counts in a replicate experiment confirmed that optogenetic treatment of MZ*oep* embryos induced fewer endodermal cells, on average, than are found in wild-type embryos (Fig. S7). Collectively, these results demonstrate that we can rescue specification of endodermal precursors and gastrulation-associated internalization movements using targeted optogenetic stimulation.

### Replacement of endogenous Nodal signaling with patterned illumination

**Fig. 4.**
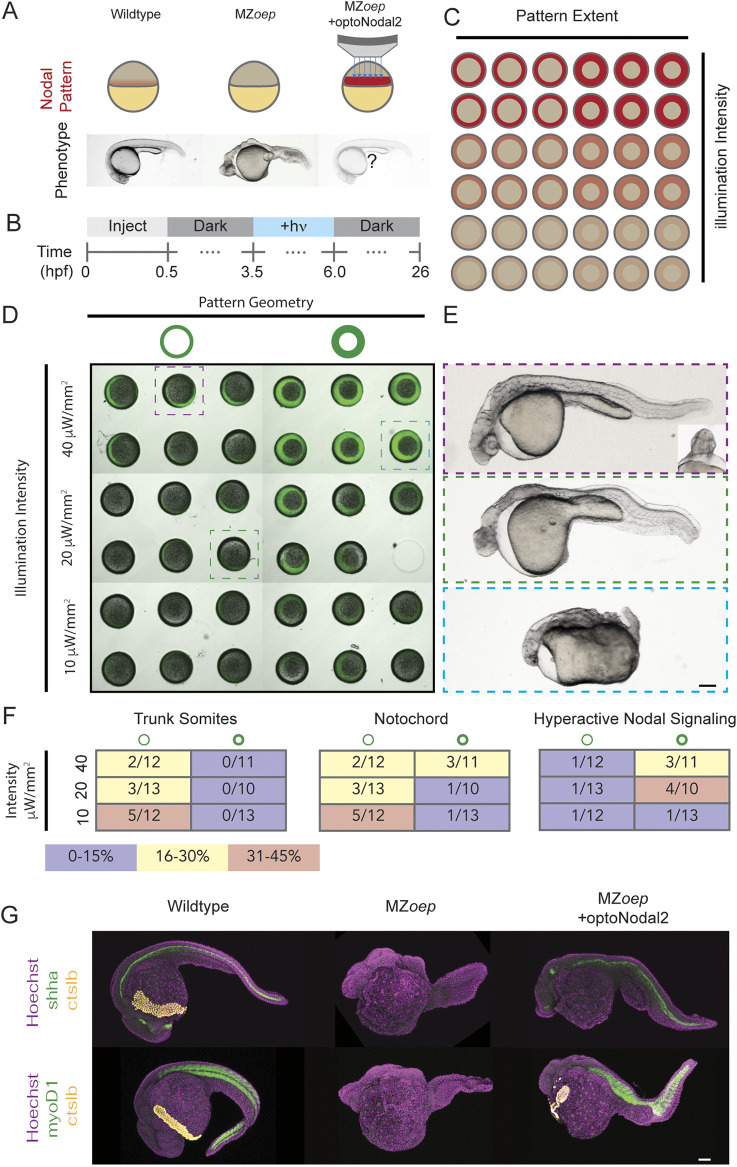
**Optogenetic rescue of Nodal signaling mutant phenotypes.** (A) Experimental overview. The absence of Nodal signaling in MZ*oep* mutants (middle) results in loss of nearly all mesendodermal tissues. We injected optoNodal2 mRNA (15 pg/receptor) into one-cell stage MZ*oep* embryos and replaced endogenous Nodal signaling with patterned optogenetic stimulation (bottom). (B) Experimental timeline. Illumination patterns were applied from 3.5 to 6 hpf with the indicated powers. Embryos were imaged or fixed at 26 hpf. (C) Schematic of arrayed layout of Nodal patterns. Optogenetic pattern characteristics were varied along each axis of the embryo array; pattern geometry was varied left-to-right, and pattern intensity was varied top-to-bottom. (D) Visualization of stimulation patterns. Applied patterns (green) were visualized by projecting pattern masks with 560 nm illumination and observing fluorescence from a co-injected mCherry mRNA. Each combination of pattern geometry and intensity was tested in five or six replicate embryos in the depicted experiment. The 26 hpf phenotypes of boxed embryos are highlighted in C. (E) Example rescue phenotypes. Example of a partial rescue phenotype (top), exhibiting notochord, trunk somites and partial rescue of cyclopia. Weaker intensity stimulation (middle) resulted in weaker rescue, with incomplete specification of trunk somites and notochord. Combining high intensity and large-area stimulation led to phenotypes reminiscent of Nodal gain-of-function (bottom, e.g. *lefty1;lefty2* double mutants). Scale bar: 100 µm. (F) Quantification of rescue phenotype frequencies for trunk somites (left), notochord (middle) and embryo disruption due to hyperactive Nodal signaling (right). Phenotypes were assessed by visual inspection of transmitted light images. (G) Visualization of marker gene expression for Nodal-dependent tissues. Top row: expression of notochord (*shha*, green) and hatching gland (*ctslb*, yellow) markers. Bottom row: expression of somite (*myod1*, green) and hatching gland (*ctslb,* yellow) markers. Scale bar: 100 µm.

We next tested whether our patterning platform could be used to induce formation of more complex Nodal-dependent tissues. An attractive application of developmental optogenetics is to test which features of morphogen signals are required for downstream development. For example, a recent study in *Drosophila* demonstrated the ability to rescue the development of a lethal patterning mutant using surprisingly simple, optogenetically evoked spatial patterns of ERK signaling (Johnson et al., 2020). The capacity to pattern many embryos simultaneously could extend this approach to systematic investigation of how features of spatial patterns encode developmental phenotypes. We therefore tested the ability of a family of stimulation patterns with a range of intensities and spatial extents to rescue the development of MZ*oep* mutants (Fig. 4A,B). We injected MZ*oep* mutants with RNA encoding optoNodal2 reagents and arrayed 36 embryos in our embryo mounts with animal pole facing the microscope objective. We illuminated each embryo with a ‘ring’ pattern that covered the Nodal signaling domain around the embryo margin (Fig. 4C). Pattern characteristics were varied along each dimension of the array; the ring width was varied along one axis (75 µm or 150 µm) and illumination intensity was varied along the other (40 µW/mm^2^, 20 µW/mm^2^ or 10 µW/mm^2^ average intensity (Fig. 4D). Embryos were stimulated from immediately before the normal onset of Nodal signaling (3.5 hpf) until the onset of gastrulation (6 hpf) to mimic the physiological duration of Nodal signaling. Embryos were collected and raised until 26 hpf in the dark, at which point they were imaged for gross phenotypes.

Our treatments elicited different Nodal phenotypes, ranging from a typical MZ*oep* phenotype (Fig. 4E, middle) to partial rescue of complex structures (Fig. 4E, top) to phenotypes consistent with Nodal gain of function [e.g. *lefty1;lefty2* double mutants (Rogers et al., 2017), Fig. 4E, bottom]. The frequency of these phenotypes correlated with the characteristics of the applied patterns (Fig. 4F). For example, patterns with lower intensity resulted in higher frequencies of MZ*oep*-like phenotypes (Fig. 4E, middle), whereas thick intense rings of illumination generated Nodal gain-of-function phenotypes (Fig. 4E, bottom). Complex structures were most often rescued with narrow, low-intensity rings of Nodal activation. In the best examples of rescue, we observe rescue of mesodermally derived structures, such as prechordal plate, notochord and trunk somites (Fig. 4E, top). We confirmed the presence of these tissues in rescued 24 hpf embryos by staining for expression of marker genes for notochord (*shha*), trunk muscle (*myod1*), and hatching glad [*hgg1* (*ctslb*)], a derivative of the prechordal plate (Fig. 4G). In some embryos, beating heart tissue was observed at the embryonic midline (Movie 2). Finally, with low frequency (2/71 embryos in two replicate experiments), we observe partial rescue of cyclopia, another hallmark of Nodal loss-of-function mutants (Fig. 4E top, inset). Collectively, these results show that development of complex tissues can be initiated by patterned optogenetic activation of Nodal signaling.

## DISCUSSION

In summary, we report the design and application of new optoNodal reagents with reduced dark activity and improved response kinetics. We combine these optoNodal2 reagents with a versatile ultra-widefield optical patterning platform to exert precise control over Nodal signaling activity in time and space, across multiple embryos in parallel. Our pipeline enables optogenetic patterning and subsequent fluorescence imaging of live zebrafish embryos with a substantial improvement in throughput over standard approaches in developmental optogenetics. We demonstrate spatial control of Nodal signaling activity and target gene expression, patterning of endodermal progenitor specification and internalization movements, and phenotypic rescue of Nodal signaling mutants. To our knowledge, the MZ*oep* rescue represents the first application of patterned optogenetics to rescue a mutant phenotype in a vertebrate embryo.

We believe that these improvements will enable optogenetic investigation of new questions requiring stringent spatiotemporal control over Nodal signaling. Indeed, an accompanying study applies our optogenetic system to reveal how Nodal signaling dynamics control convergence and extension movements of the zebrafish mesoderm (Emig et al., 2025). Future work could extend the utility of the reagents and platform we present here. For example, previous studies demonstrated two-photon activation of Cry-Cib interactions (Kennedy et al., 2010). Two-photon activation of optoNodal2 could offer a route to three-dimensional control of Nodal signaling patterns, something not achievable with our DMD-based system. Combinations of spectrally orthogonal photodimerizing proteins might allow multiple signaling pathways to be controlled independently. The rapidly expanding optogenetic toolbox across the optical spectrum could contribute to this goal. Finally, we anticipate that zebrafish lines stably expressing optoNodal2 receptors would obviate the inherently variable process of mRNA injection and could improve patterning precision and reproducibility.

A gradient of Nodal signaling has long been recognized to orchestrate mesendodermal patterning in vertebrate embryos. At first glance, experiments from zebrafish (Chen and Schier, 2001; Dougan et al., 2003; Gritsman et al., 2000; Schier et al., 1997; Thisse et al., 2000) make a compelling case for a concentration threshold-like model; high, medium and low concentrations of Nodal correlate with spatially ordered populations of endoderm, mesoderm and ectoderm, respectively. However, several experimental observations suggest a more-complex picture. The duration of Nodal exposure (Gritsman et al., 2000; Hagos and Dougan, 2007), timing of signal onset and cessation (Sako et al., 2016), speed of Nodal spread through space (van Boxtel et al., 2018) and kinetics of target gene transcript accumulation (Dubrulle et al., 2015) have all been shown to influence fate selection. Complicating matters further, a recent study suggested that Nodal-mediated endoderm fate selection is probabilistic (Economou et al., 2022). Our recent observations (Lord et al., 2021) suggest that a surprising degree of patterning can be achieved even without a stable gradient. In zygotic *oep* mutants – a background that successfully specifies somites and notochord (Schier et al., 1997) – the Nodal gradient is transformed into a wave of signaling activity that propagates from the margin toward the animal pole (Lord et al., 2021). We do not yet know what constraints the Nodal pattern needs to satisfy or how spatial or temporal features of Nodal signaling allow the embryo to meet them. We anticipate that the approaches we present here will prove useful for answering these questions by enabling Nodal pattern features and dynamics to be manipulated precisely.

Modeling efforts have aimed to explain how diffusion and capture give rise to morphogen profiles (Crick, 1970; Kerszberg and Wolpert, 1998; Müller et al., 2012, 2013; Wartlick et al., 2009; Yu et al., 2009), or how cells transform continuously varying concentrations into discrete fate choices (Wolpert, 1969). An enduring challenge with these efforts is that a lack of variation in observed morphogen profiles leaves the models underdetermined. To rigorously constrain quantitative models, we need access to rich libraries of morphogen signaling profiles. For example, models would benefit from datasets that systematically varied signaling gradient range, shape, rate of change and orientation. In the rare cases where such manipulation is possible, surprising outcomes are common. For example, flattened Bicoid gradients perform remarkably well in patterning target gene expression (Ochoa-Espinosa et al., 2009), despite the fact that traditional concentration-centric models predict marked shifts in expression domain boundaries. By making it possible to generate libraries of complex signaling patterns in dozens of embryos simultaneously, we believe that the tools presented here will facilitate rigorous testing of quantitative models.

## MATERIALS AND METHODS

### Zebrafish husbandry

Zebrafish (*Danio rerio*) were raised and maintained according to standard practices (Westerfield, 2020). Briefly, embryos were grown in embryo medium (250 mg/l Instant Ocean salt in distilled or reverse osmosis-purified water, adjusted to pH 7.0 with NaHCO_3_) supplemented with 1 mg/ml Methylene Blue. Wild-type breeding stocks were the result of TL×AB crosses (TL and AB stocks were obtained from ZIRC). *vg1* and *oep* mutant fish were propagated as previously described (Gritsman et al., 1999; Montague and Schier, 2017). Staging was performed using a combination of time measurement (i.e. time elapsed since fertilization) and morphological examination as compared to a standard staging series (Kimmel et al., 1995). M*vg1* mutant embryos were obtained by mating *vg1^−/−^*females to TLAB wild-type males. MZ*oep* mutant embryos were obtained by incrossing *oep*^−/−^ adult fish. All animal experiments were performed under the supervision of the University of Pittsburgh IACUC (protocol ID 23124380).

### mRNA synthesis and embryo microinjection

Coding sequences for mRNAs used in this study (*myr-acvr1b-cry2*, *acvr2b-cibn*, *myr-acvr1b-LOV* and *myr-acvr2b-LOV*) were cloned into pCS2+ vectors. Briefly, *myr*-*acvr1b-lov* and *myr*-*acvr2b-lov* transcription templates were a gift from the Heisenberg Lab (Sako et al., 2016), and Cry2- and Cib1N-coding sequences were obtained from Addgene (Addgene plasmid #26866, deposited by Chandra Tucker; Addgene plasmid #26867, deposited by Chandra Tucker, respectively). We replaced the LOV domain sequences in the Myr-Avr1b-LOV- and Myr-Acvr2b-LOV-coding sequences with Cry2 and Cib1N, respectively, using Gibson assembly cloning. The myristoylation motif was removed from the Acvr2b construct by ‘round the horn PCR’ (PCR amplifying the entire plasmid – omitting the myristoylation motif – with phosphorylated primers and subsequent ligation to recircularize). To transcribe mRNA, plasmid templates were linearized with NotI and purified using Monarch PCR purification kits (New England Biolabs). The purified templates were transcribed using mMESSAGE mMACHINE Sp6 (Thermo Fisher Scientific) kits. mRNAs were purified using the Monarch RNA Cleanup Kit (New England BioLabs) and eluted in RNAse-free water. All kits were used according to the manufacturer's specifications. Plasmids encoding optoNodal2 reagents were deposited with Addgene (Addgene plasmid #161715 and Addgene plasmid #161720).

mRNA microinjections were carried out using Drummond Nanoject III injector instruments. Injections were performed directly into the blastomere of 1-cell stage dechorionated embryos. Injections were typically 2.0 nl in volume, and embryos raised in agarose-coated six-well dishes in embryo medium supplemented with Methylene Blue following injection. All embryos injected with optogenetic reagents were kept in aluminum foil-wrapped plates for all timepoints after 2.0 hpf.

### OptoNodal Receptor light intensity and impulse response measurements

All intensity response and kinetic response measurements from Fig. 1 were obtained using the open-source optoPlate-96 instrument (Bugaj and Lim, 2019) (dual blue LED configuration). Our instrument was fabricated by the machine shop at the University of Pittsburgh Department of Cell Biology. Power calibration for each LED was performed using a ThorLabs PM100d power meter and a custom MATLAB analysis script. Experiments were designed – with intensity correction factors applied – and transferred to the optoPlate Arduino processor using the OptoConfig software package (Thomas et al., 2020). For the light intensity response series (Fig. 1C,D), we injected 15 pg of either Cry-Cib or LOV optoNodal reagents into M*vg1* embryos at the one-cell stage. A 1 h light treatment (average powers of 1,5,10,25 and 50 µW/mm^2^) was initiated at sphere stage. These light pulses consisted of a 33% duty cycle (10 s on, 20 s off) with instantaneous powers of 3, 15, 30, 75 and 150 mW/µm^2^. All embryos were harvested and immediately fixed overnight at 4°C in 4% formaldehyde in 1× PBS. For the impulse response measurements (Fig. 1E,F), we injected 15 pg of either Cry-Cib or LOV optoNodal receptors RNAs (i.e. 15 pg each of type I and type II receptors) into M*vg1* embryos at the one-cell stage. A 20 min light treatment with average power 20 µW/mm^2^ (60 µW/mm^2^ instantaneous power with 33% duty cycle) was applied beginning at dome stage. At the indicated times, embryos were harvested and fixed overnight at 4°C in 4% formaldehyde in 1× PBS. For both experiments, fixed embryos were immunostained for pSmad2. For the replicate impulse response measurements presented in Fig. S2, we injected 15 pg of either Cry-Cib or LOV optoNodal receptors RNAs (i.e. 15 pg each of type I and type II receptors) into MZ*oep* embryos at the one-cell stage. Fixation and immunostaining were performed as described for the impulse response experiments of Fig. 1.

### Fixed embryo staining, imaging and quantification

α-pSmad2 immunostaining was performed as previously described (Lord et al., 2021). The primary antibody used was CST 18338 (RRID:AB_2798798) at 1:1000 dilution. Antibody specificity was previously verified with negative control staining in Nodal loss-of-function mutants (Lord et al., 2021). *noto*, *lft2*, *shha*, *sox32* and *myod1* transcripts were detected using an HCR 3.0 protocol (Choi et al., 2018). HCR staining was carried out according to manufacturer instructions for <1 dpf zebrafish embryos. Our α-*flh* and α-*lft2* probesets were visualized with AlexaFluor 647-conjugated B3 and AlexaFluor 488-conjugated B2 HCR 3.0 hairpins, respectively. Probes for *shha* and *myod1* were visualized using AlexaFluor 546-coupled B2 hairpins, and *sox32* probes were visualized with AlexaFluor 647-coupled B3 hairpins. Both HCR and pSmad2-stained embryos were mounted in 1% low-melt agarose and imaged on Nikon A1 laser scanning confocal microscopes at the University of Pittsburgh Center for Biological Imaging. *Z*-stacks were acquired with a 2.5 µm spacing on either 10× or 20× air objectives.

α-pSmad2 staining intensity was quantified using a custom MATLAB image analysis pipeline described previously (Lord et al., 2021). Briefly, Sytox green-stained nuclei within 25 µm of the embryo animal pole were segmented using a combination of local adaptive thresholding, morphological filtering and active contours boundary refinement. Automated segmentation results were further refined by manual inspection and correction with a custom MATLAB interface. Fluorescence intensities on each imaged channel were compiled for each segmented object. For the quantification panels in Fig. 1, mean pixel intensities within each mask were used. Statistical comparisons between mean intensities of different conditions (e.g. background comparisons in Fig. S1) were performed using an unpaired sample *t*-test.

As noted in the legend of Fig. 1, we observe variation in illumination responses within individual embryos. This variability likely represents imperfect dispersal of optoNodal or optoNodal2 mRNA after microinjection. We elected to include all segmented nuclei in the presented quantifications, rather than attempt to exclude nuclei from cells with putatively low mRNA dosages. We believe this to be the most rigorous practice for two reasons. First, mRNA distribution is uncorrelated with the choice of construct being injected. These errors therefore did not introduce a systematic bias. Second, thresholding based on the quantity we are attempting to measure (i.e. pSmad2 staining intensity) undercounts cells with low expression levels.

### Embryo mount design and fabrication

Molds for embryo ‘egg crate’ mounts were designed using TinkerCad software. Mounts were arrays of short, embryo-sized ‘posts’ with varying radii (275, 300, 325 and 350 µm) and height 600 µm that created individual wells for embryos when molded. Four corner posts of height 3 mm set the spacing between the bottom of the dish and the wells. Each design was exported as toolpath (.stl) files and printed using a Form 3 SLA printer (Formlabs). Some variation in feature dimensions occurs between prints, so the appropriate mold should be selected empirically in a pilot experiment.

To mold embryo eggcrate mounts, 3.0 ml of melted 0.5% agarose in embryo medium was dispensed into a well of a six-well polystyrene tissue culture plate. The 325 µm egg crate mount mold was placed into the agarose, and excess agarose was removed, to allow the corner spacer legs to contact the bottom of the dish. Mounts were allowed to solidify at 4°C for >1 h, and the mold was manually removed using a scalpel. For patterning experiments, 1-2 ml 0.2% low-melt agarose was layered over the egg crate mount, and embryos were manually loaded into the well array and oriented. The low-melt agarose overlay was allowed to gel for ∼15 min at room temperature before mounted embryos were moved to the microscope for optical patterning experiments.

### Patterning endoderm internalization

MZ*oep* embryos were injected with a total of 30 pg of mRNA encoding each optoNodal2 receptor and 150 pg of mCherry mRNA at the four-cell stage (each blastomere was injected with 0.5 nl of the mRNA mixture). Embryos were grown in the dark in embryo medium until 3.0 hpf at 28.5°C, at which point they were transferred into embryo array mounts as described above. Optogenetic treatments were carried out from 3.75-6.25 hpf. Stimulation patterns comprised annular rings at the embryo margin of 75 µm thickness with instantaneous intensity of 240 µW/mm^2^. With scanning over six positions, this resulted in an average intensity of 40 µW/mm^2^ (i.e. 16% duty cycle with 20 s dwell time at each position). After stimulation, embryos were immediately retrieved from array mounts and fixed overnight in 4% formaldehyde at 4°C in the dark. Fixed embryos were stained for *sox32* expression using HCR as described above. Embryos were counterstained with Hoechst nuclear stain. Stained embryos were mounted with the A-V axis parallel to a No. 1.5 glass coverslip in 1% low-melt agarose and imaged on a Nikon A1 confocal with 20× objective on Hoechst and Alexa647 channels. *Z*-stacks were obtained with 5 µm between slices; presented images are maximum intensity projections.

### Optogenetic rescue of MZ*oep* mutant phenotype

Four-cell MZ*oep* embryos were injected with an mRNA cocktail containing mCherry and optoNodal2 receptors. Each embryo received a total of 15 pg of each receptor and 150 pg of mCherry mRNAs. Embryos were grown in the dark in agarose-coated six-well plates containing embryo medium until 3 hpf, at which point they were transferred to embryo array mounts for patterning. Optogenetic stimulation was performed using the mask array depicted in Fig. 4. The average powers of 40, 20 and 10 µW/mm^2^ indicated in the figure were achieved using instantaneous intensities of 240, 120 and 60 µW/mm^2^, respectively (a total of six positions were scanned cyclically with a 20 s patterning dwell at each position). To visualize pattern registration, transmitted light (using a 635 nm ‘safe light’ LED positioned under the stage) and fluorescent images (using patterned illumination on the RFP channel) were taken every 15 min. After patterning, each embryo was transferred to a well of an agarose-coated 24-well plate containing embryo medium. Transfers were performed to preserve the ordering of embryos in the patterning array; that is, each embryo phenotype could be directly connected back to the live images taken during patterning. Phenotypes were assessed at 26 hpf by mounting embryos laterally in 2.2% methylcellulose and transmitted light imaging on a Leica M165 FC upright microscope. Tissue marker gene expression (e.g. *shha*, *ctslb*, and *myod1*) was visualized by HCR 3.0, as described above.

### Patterning microscope design

Experiments were performed on two versions of an ultra-widefield patterning microscope. Preliminary experiments were performed on a custom-built design (the ‘Firefly’) described previously (Farhi et al., 2019). For data shown in this study, we reproduced a Firefly-like microscope using more accessible commercial components. The core of our patterning system was built around a Mightex OASIS Macro DMD microscope. This core system comprised an array of LEDs (405 nm, 470 nm, 560 nm and 625 nm) that were routed to a DMD projector (Mightex Polygon 1000, 1140×912 pixels) via a liquid light guide and a 0.37 NA objective macro lens. The overall magnification of the projection path was 2×, yielding an effective projection ‘pixel size’ of 3.8 µm at the sample plane. The overall imaging of the imaging path of the system is 4×. To facilitate our experiments, we made the following modifications to the system.

#### Camera

To facilitate rapid, high-sensitivity imaging, we installed a Hamamatsu Orca Fusion III sCMOS camera in the observation path. The camera was triggered using custom software (see below) via voltage pulses from an Arduino controller through the external trigger port.

#### Objective lens

To control the angular content of incident patterned light, we contracted with Mightex to install a movable iris at the back focal plane of the objective lens. By closing this aperture, the angular content of patterned light could be reduced, resulting in the ‘pencil beam’ configuration used in most patterning experiments in this study. This feature was included in order to render projected patterns less sensitive to the position of an embryo with respect to the focal plane of the objective.

#### Filter wheel and main dichroic

To enable multi-channel imaging without channel crosstalk, we installed a large aperture (50 mm) motorized filter wheel (Edmund Optics, 84-889) with DAPI, GFP, RFP and E2-Crimson band emission filters (Chroma). The filter wheel was inserted into the light path using a custom-machined threaded adapter. We replaced the 50-50 beam splitter in the original Oasis Macro design with a large-area, 4-band dichroic (Semrock, DIO3-R405/488/561/635-t3). To minimize pattern distortion due to dichroic curvature over its large area, we selected a 3 mm-thick, 42×60 mm material. To fit the dichroic into Macro beam splitter housing, we milled ∼1 mm of excess material out of the Mightex dichroic housing.

#### Motorized stage

To enable automated scanning between multiple positions, we installed a motorized XYZ encoded stage (Prior Instruments, H101E1F XY motor with FB206 focus block stage and ProScan III Controller). Both XY position and Z focal control were managed by moving the sample in three dimensions with the stage.

#### Sample incubation

Sample temperature and humidity were controlled during experiments using an OkoLabs BoldLine stage-top incubator system with active humidity and temperature control. Since the microscope has an upright design, patterning and imaging were performed through a transparent lid with active heating to prevent condensation during long experiments.

#### Microscope control

All experiments were performed using a custom-built interface coded in MATLAB. This interface consisted of a custom GUI to streamline: (1) DMD calibration, (2) multi-position selection and (3) imaging setting selection. Patterning experiments were performed using custom MATLAB scripts. Our software interface was designed with object classes for the camera, stage, LED array and DMD instruments. Each object class was designed with high-level class methods to execute hardware commands (e.g. move stage to XYZ position, capture image, activate LED, etc.). Acquisition scripts were built using these high-level methods. The software interface used here was custom-developed in the Lord lab. An open-source software package for control of DMD microscopes (‘Luminos’) has also been recently developed and release by the Cohen lab (Itkis et al., 2025 preprint).

#### Patterned illumination

The light projection path of he microscope was calibrated before each patterning or acquisition session. To register the coordinates of the DMD projector with spatial coordinates at the sample plane, we projected and imaged a mask containing 10 circular spots with known centroid positions onto a microscope slide with a mirrored surface. This image was then used to fit an affine transformation that maps DMD coordinates to sample plane coordinates. This transform was used to ensure that each projected pattern was properly registered to the targeted spatial coordinates on the sample. To correct an uneven illumination intensity profile, it was measured using static illumination with all pixels ON and imaging its reflection on a mirror. This profile was proportionally applied as the grayscale value of projected masks to achieve a uniform illumination intensity across the FOV. Illumination intensity was measured automatically using a power meter (Thorlabs PM100D), before each experiment at the relevant LED currents.

## Supplementary Material



10.1242/develop.204506_sup1 Supplementary information
