# Supplementary material for: Optogenetic control of Nodal signaling patterns
Source: Development. 2025 May 1;152(9):dev204506. doi: 10.1242/dev.204506 (PMC12070070; doi:10.1242/dev.204506)
Supplement: Supplementary information [file develop-152-204506-s1.pdf]

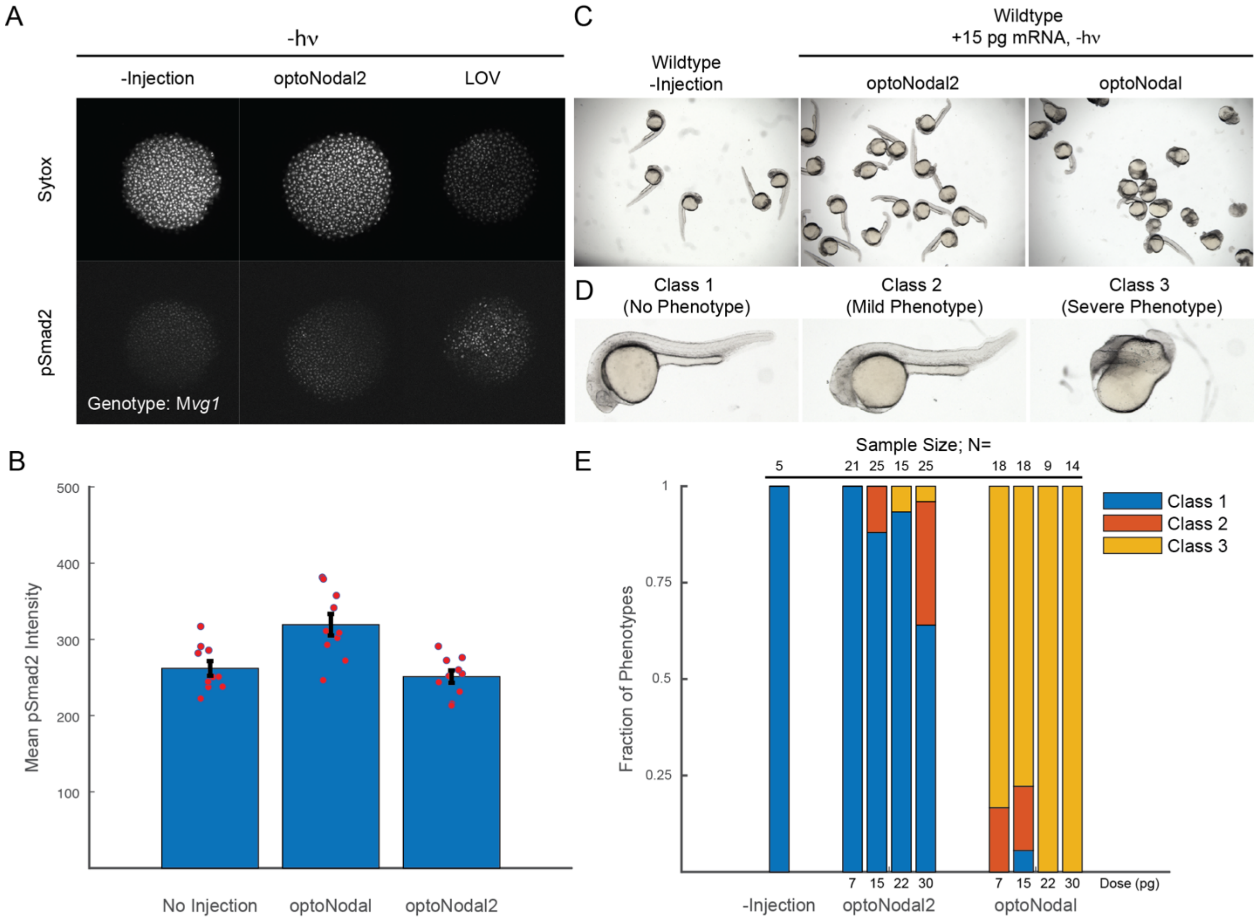

Fig. S1. Comparison of dark activity in optoNodal vs. optoNodal2 reagents. (A) Visualization of dark activity. *Mvg1* embryos were unperturbed ('-Injection') or injected with mRNA encoding Cry-Cib-based optoNodal2 or LOV-based optoNodal receptors (15 pg per receptor mRNA). Embryos were raised in the dark until 6 hpf, fixed and immunostained for α-pSmad2 (bottom row). (B) Quantification of α-pSmad2 staining intensity in unilluminated embryos. Graph depicts mean α-pSmad2 nuclear staining intensity, and error bars denote s.e.m.. Statistical comparisons between samples were performed with an unpaired sample t-test with asterisks denoting $p < 0.05$. (C) Representative 24 hpf phenotypes of wild-type embryos injected with 15 pg of Cry-Cib or LOV-based optoNodal receptors. (D) Example images denoting phenotypic classes quantified in panel E. Class I embryos exhibit no gross abnormalities, Class II embryos exhibit loss of head structures and/or pronounced axis curvature, Class III embryos exhibit severe dorsalization consistent with excess Nodal signaling activity. (E) Distribution of embryos between phenotypic classes in wild-type embryos without injection or injected with indicated amounts of optoNodal2 or optoNodal receptor mRNAs.

Development • Supplementary information

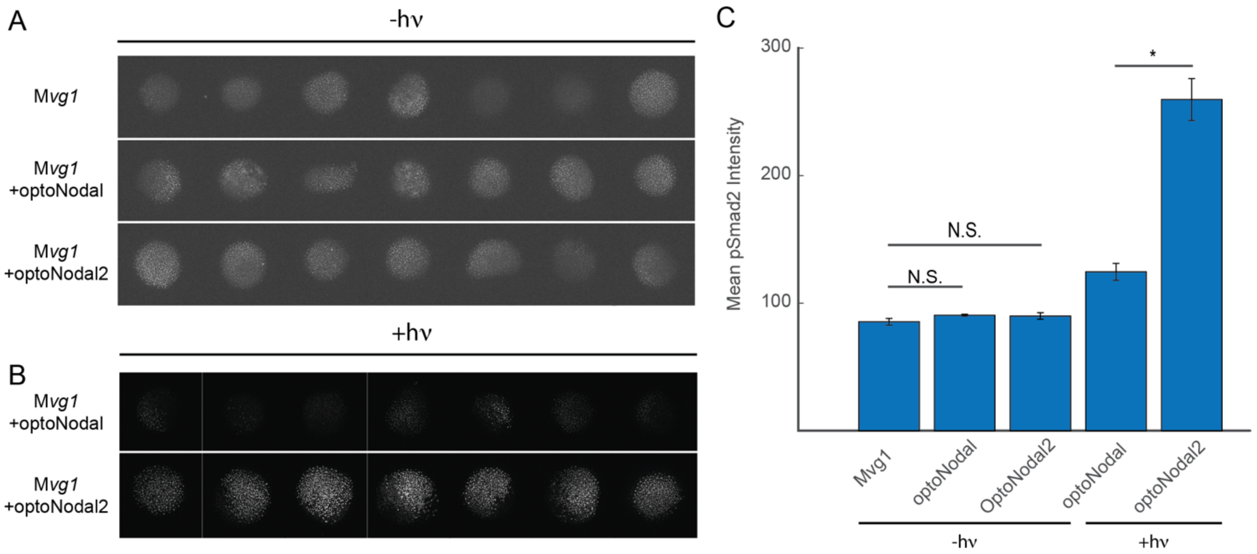

Fig. S2. Comparison of optoNodal and optoNodal2 dynamic range at background-matched dosage. Fig. 1 of the main text compares optoNodal and optoNodal2 at equal mRNA doses. Here, we compare optoNodal and optoNodal2 at 'functionally matched' dose, i.e. the highest dose of each receptor that does not lead to adverse phenotypes at 24 hpf (3.75 pg for optoNodal, 15 pg for optoNodal2). (A) Dark activity. *Mvg1* embryos were (top) unperturbed ('M*vg1*') or injected with mRNA encoding (middle) LOV-based optoNodal or (bottom) Cry-Cib-based optoNodal2 receptors. Embryos were raised in the dark until 5 hpf, fixed and immunostained for pSmad2. Each image represents a maximum intensity projection of 10 confocal slices. (B) Visualization of maximal activity. OptoNodal and optoNodal2-injected M*vg1* embryos were illuminated with a saturating dose of light (50 $\mu W/mm^2$ average power) for 1 hour beginning at 4 hpf. After treatment, embryos were fixed and immunostained for pSmad2. Each image represents a maximum intensity projection of 10 confocal slices. Image contrast is shared across all images in (B), but is distinct from images in (A). (C) Quantification of α-pSmad2 staining. Graph depicts mean α-pSmad2 nuclear staining intensity, and error bars denote standard error of the mean (n = 7 embryos per condition). Statistical comparisons between samples were performed with an unpaired sample t-test with asterisks denoting $p < 0.05$.

Development • Supplementary information

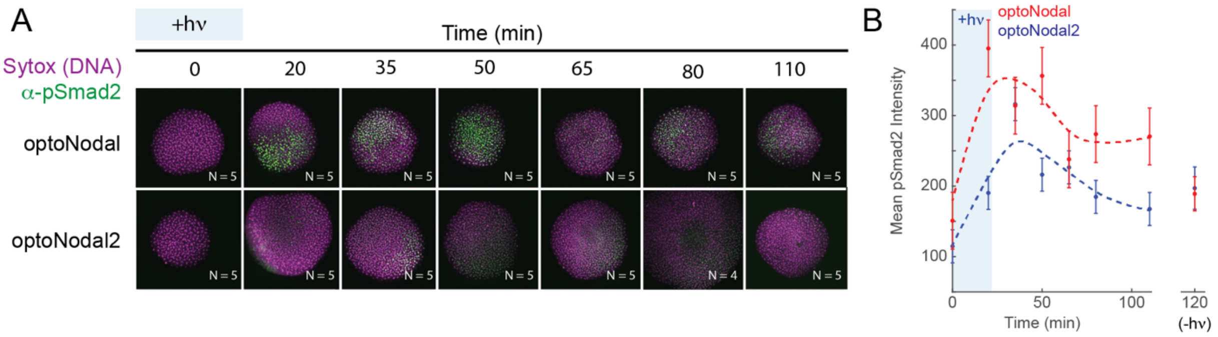

Fig. S3. Dynamic responses of optoNodal and optoNodal2 in MZ*oep* mutants. To confirm the observations of Fig. 1 E,F, the responses of optoNodal and optoNodal2 reagents to a 20 minute impulse of light were measured in MZ*oep* mutant embryos. (A) Measurement of response kinetics for optoNodal (top row) and optoNodal2 (bottom row) reagents. Embryos injected with indicated reagents (15 pg per receptor mRNA) were illuminated for 20 minutes with 470 nm light beginning at dome stage. Embryos fixed after the indicated delays and Nodal signaling was measured by α-pSmad2 immunostaining (green). Images are maximum intensity projections of representative embryos. (B) Quantification of Nodal signaling activity from panel A. α-pSmad2 staining intensity was extracted from segmented nuclei in optoNodal (red) and optoNodal2 (blue) treatment groups; each point represents the average nuclear staining intensity from the indicated number replicate embryos in Panel A. Error bars denote the standard error of the mean. Background intensity of unilluminated embryos at the 120 minute timepoint are included (-hν) to indicate baseline levels of signaling activity.

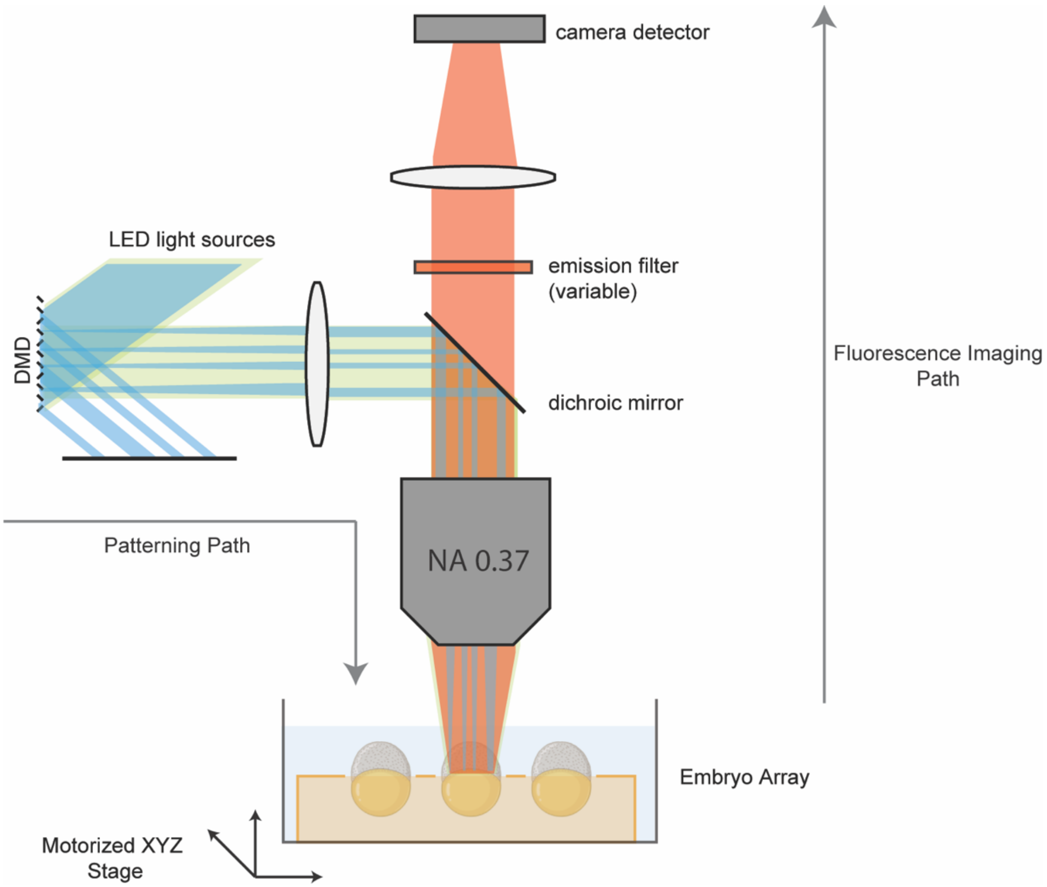

Fig. S4. Design of spatial patterning microscope. Our platform is a modified version of the 'Firefly' microscope design described by Werley *et al* (Werley *et al.*, 2017) and modified by Farhi *et al* (Farhi *et al.*, 2019). We modified an Oasis Macro ultra-widefield patterning microscope from Mightex. To create spatial patterns at the sample plane ('Patterning Path'), light from a multi-color LED illuminator is directed to the face of a DMD using a liquid light guide. Pixels on the DMD have two states, 'ON' and 'OFF', with 'ON' pixels directing light toward the sample. Pattern masks are encoded as pixel states on the DMD, and patterned light is collected by a projection lens, reflected of of a multi-band dichroic mirror, and reimaged onto the sample plane using a 4x 0.37 NA objective lens. Emitted light from the sample is collected ('Fluorescence Imaging Path') by the objective lens, passed through the dichroic mirror and a wide-format emission filter on a motorized wheel, and reimaged onto a Hamamatsu Orca Fusion III sCMOS camera by a tube lens. Magnification along the projection path (i.e. from DMD to sample plane) is 2x. Magnification along the imaging path (i.e .from sample plane to camera) is 2x. Sample positioning in three dimensions is controlled via an automated XYZ stage.

Development • Supplementary information

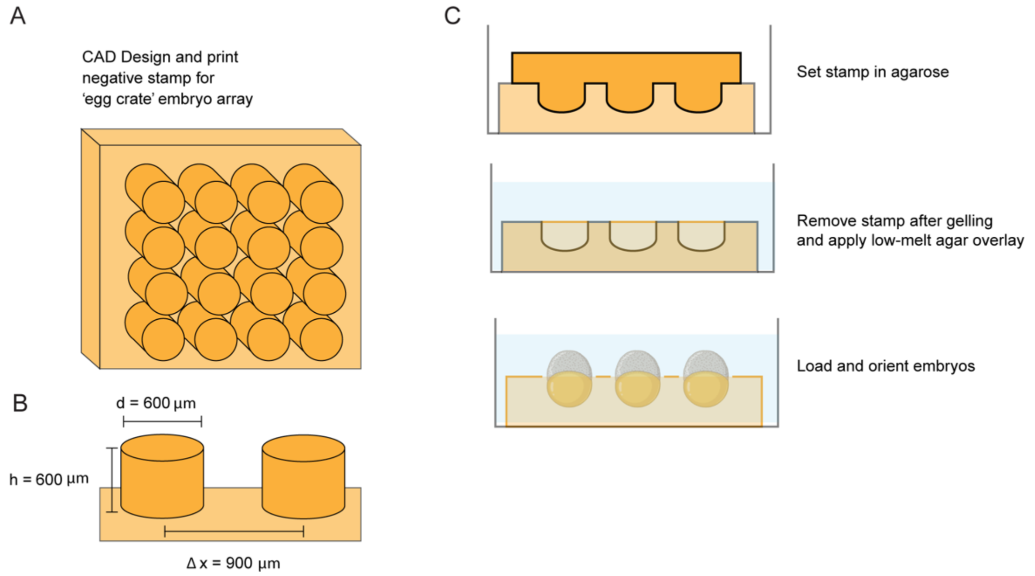

Fig. S5. Design and fabrication of embryo array mounts. (A) Design of embryo array mounts. A negative 'egg crate' stamp consisting of an array of cylindrical posts was designed using TinkerCAD. (B) Typical dimensions of embryo array stamps. For ost experiments, an array of cylindrical posts with 600 µm diameter and height (separated by 300 µm in oth dimensions) was used. Stamps were 3D printed using a Form 3 SLA printer. (C) Schematic of procedure used to generate agarose embryo mounts from 3D printed stamps. Stamps were pressed into molten 0.5% agarose in embryo medium. After setting, the stamps were manually removed, and an overlay of 0.2% low-melt agarose in embryo medium was pipetted on top at a temperature of ~42 °C. Embryos were then mounted in the devices and manually oriented before the low-melt agarose solidified. Once encased between regular and low-melt agarose, embryos were used for patterning experiments.

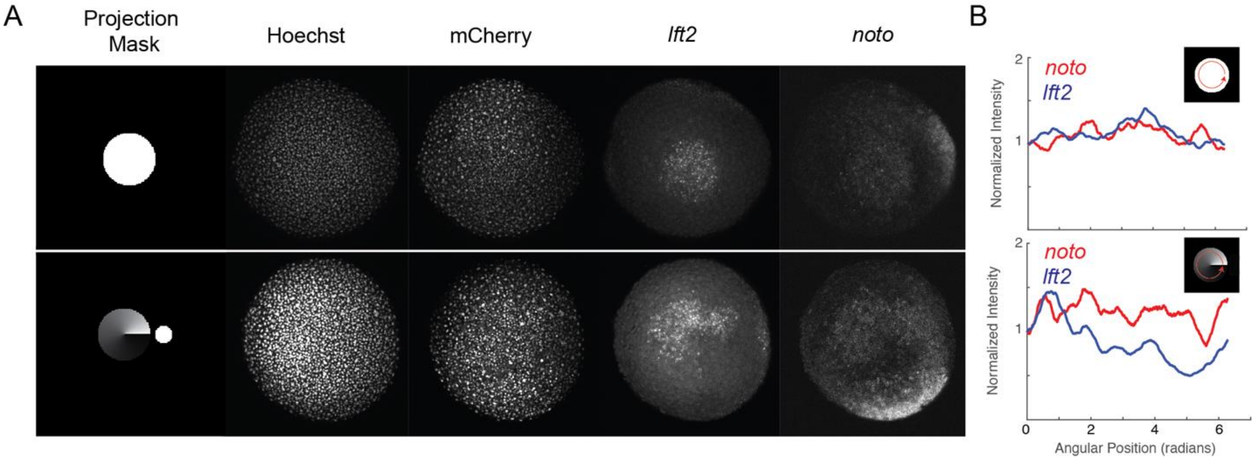

Fig. S6. Projection of graded patterns of light. The spatial patterning in Fig. 2 used binary patterns composed of ON and OFF pixels. Greyscale patterning can be achieved by modulating the pixel duty cycle at a frequency > 1 kHz, much faster than the optoNodal2 reagent response. A) Comparison of solid and graded projection patterns. Spot (top row) or radial gradient (bottom row) patterns were projected onto the animal pole of wild-type embryos injected with mRNA encoding optoNodal2 (15 pg per receptor mRNA) receptors and nuclear mCherry (100 pg). Gradient patterns also included a solid 'spot' as a fiducial to mark the position of the 'bright' position of the gradient. Patterns were applied for 45 minutes beginning at sphere stage. Peak gradient power corresponded to 20 $\mu W/mm^2$, and power decreased exponentially with an angular half-distance of 0.17 radians. Patterned embryos were fixed immediately after patterning and stained for expression of *lft2* and *noto* by hybridization chain reaction (HCR). B) Quantification of *lft2* (blue) and *noto* (red) expression in spot (top) and gradient (bottom) patterned embryos. Plots depict the average pixel intensity as a function of radial position within the gradient (see red contour in insets). The zero radians position corresponds to the brightest position in the gradient. Curves depict sliding window averages of width 0.5 radians. Both genes showed similar expression profiles in the spot pattern. In the gradient pattern, *lft2* expression decreased more sensitively with light intensity than *noto*, consistent with previous measurements of Nodal target gene characteristics (Dubrulle et al., 2015).

Development • Supplementary information

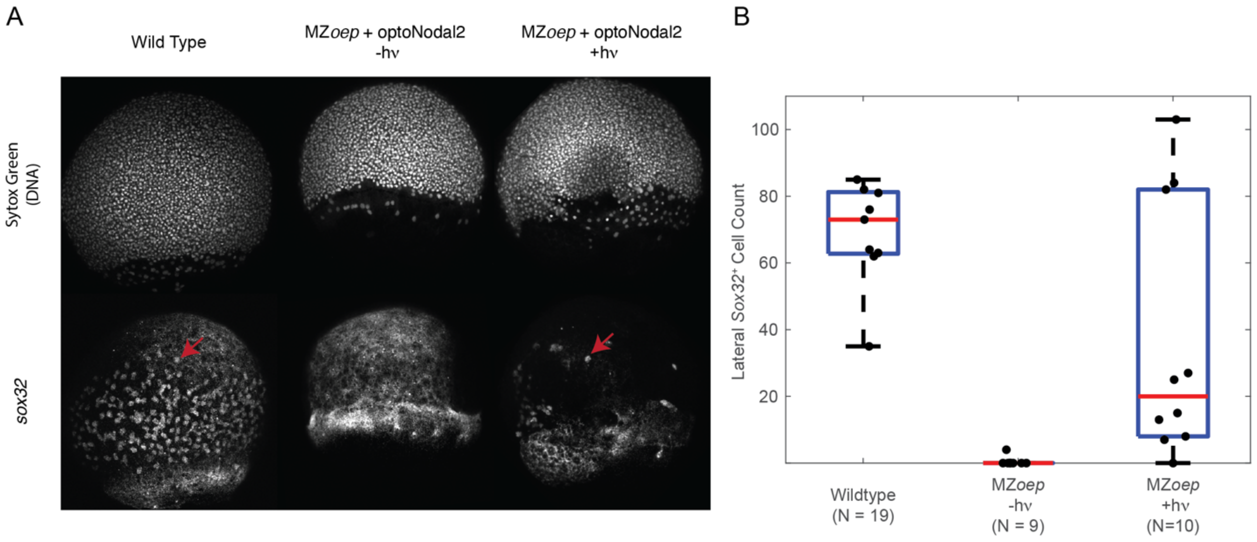

Fig. S7. Quantification of *sox32*⁺ cell induction with optoNodal2 reagents. This experiment replicates the results in Fig. 3 of the main text. Briefly, MZ*oep* mutant embryos were injected with 30 pg of each optoNodal2 receptor mRNA. Marginal ring illumination patterns were applied to '+hν' samples from 3.75 to 6.25 hpf at 40 μw/mm^2 average intensity. Embryos were fixed at 8 hpf and stained for *sox32* expression by HCR. A) Comparison of *sox32*+ cell distributions in wild-type (left column), dark control (middle column) and illuminated embryos (right column). Images depict maximum intensity projections of the lateral region of representative embryos. Red arrows highlight example *sox32*-expressing cells. B) Quantification of *sox32*$^+$ cell counts. *Sox32*-positive cells were manually counted in a 300 μm-wide window at the center of the lateral region. Red lines depict average counts, boxes depict the 25th-75th percentile range, black dots depict individual embryo cell counts. All pairwise comparisons between group means achieve statistical significance ($p < 0.05$) by unpaired sample t-test.

Development • Supplementary information

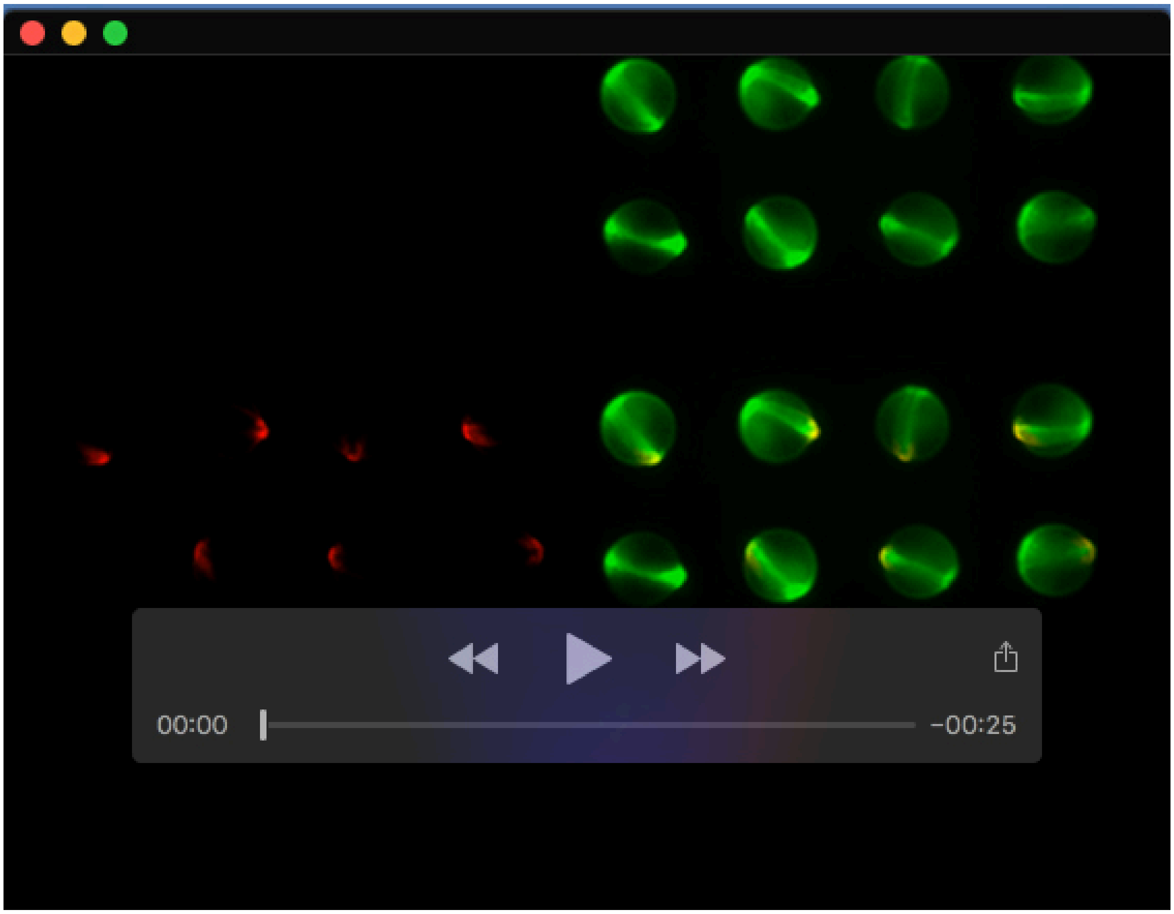

Movie 1. Direct visualization of photopatterning of live zebrafish embryos. To map optical doses delivered to the embryos, wild-type zebrafish embryos were injected at the single-cell stage with mRNA encoding a green-to-red photoconvertible fluorescent protein (Kaede). At sphere stage, embryos were stimulated with 405 nm light with the indicated spatial masks (upper left). Embryos were then imaged every 10 minutes on GFP (upper right) and RFP (lower right) fluorescent channels until 24 hpf.

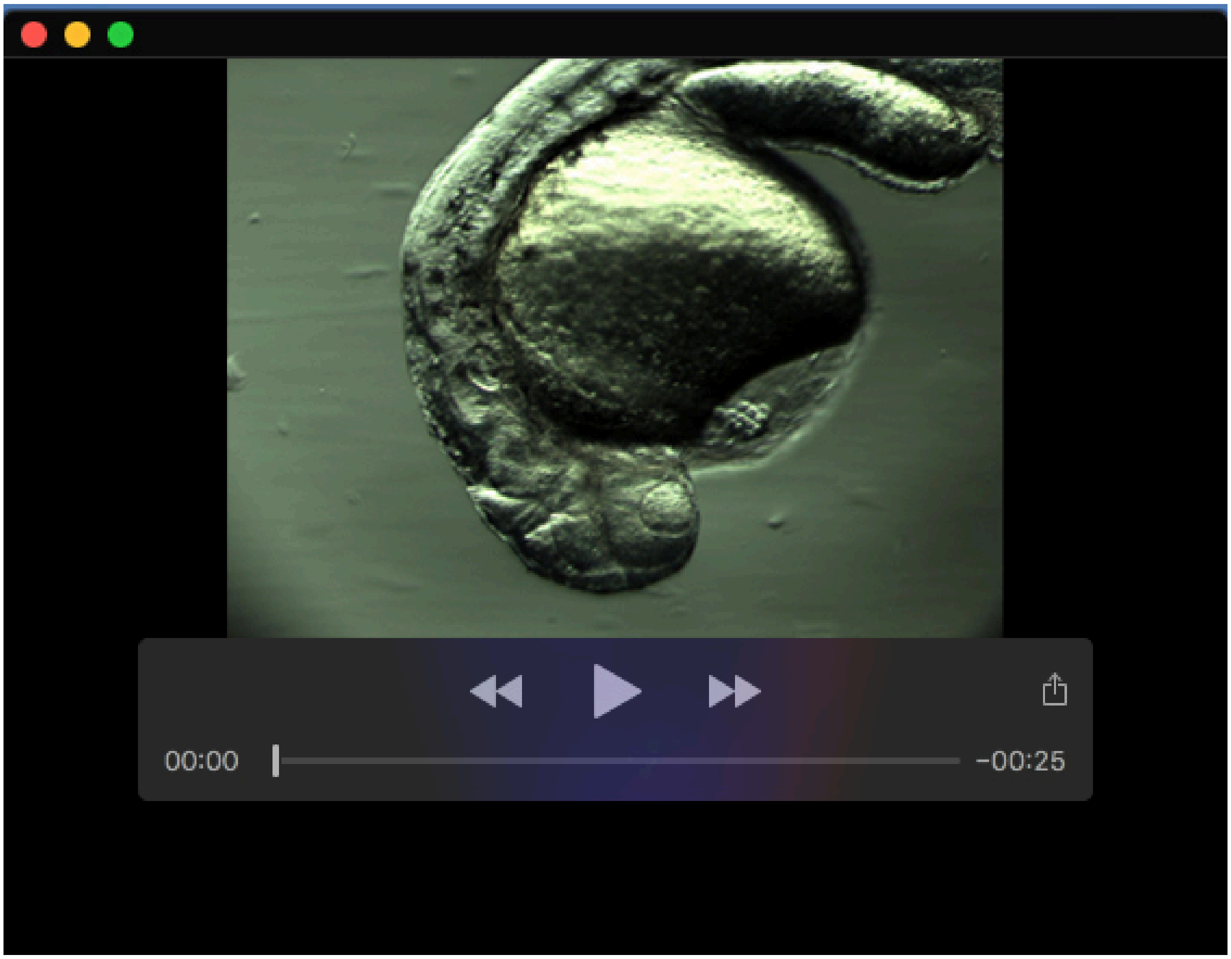

Movie 2. Visualization of beating heart tissue in an optogenetically-rescued MZoep mutant. The 26 hpf embryo was partially rescued with targeted illumination as described in Fig. 4. Beating heart tissue is located at the midline, suggesting presence of endoderm.
