# Supplementary material for: Optogenetic control of Nodal signaling patterns
Source: Development. 2025 May 1;152(9):dev204506. doi: 10.1242/dev.204506 (PMC12070070; doi:10.1242/dev.204506)
Supplement: Supplementary file 1 [file develop-152-204506_review_history.pdf]

Optogenetic control of Nodal signaling patterns

Harold M. McNamara, Alison M. Guyer, Bill Z. Jia, Vicente J. Parot, Caleb D. Dobbs, Alexander F. Schier, Adam E. Cohen and Nathan D. Lord
DOI: 10.1242/dev.204506

Editor: Steve Wilson

Review timeline

Original submission:	9 April 2024
Editorial decision:	24 May 2024
First revision received:	6 January 2025
Editorial decision:	17 February 2025
Second revision received:	18 March 2025
Accepted:	20 March 2025

Original submission

First decision letter

MS ID#: DEVELOP/2024/202911

MS TITLE: Optogenetic control of Nodal signaling patterns

AUTHORS: Harold M McNamara, Bill Z Jia, Alison Guyer, Vicente J Parot, Caleb Dobbs, Alexander F Schier, Adam E Cohen, and Nathan D Lord

I have now received all the referees reports on the above manuscript, and have reached a decision. The referees' comments are appended below, or you can access them online: please go to BenchPress and click on the 'Manuscripts with Decisions' queue in the Author Area.

The reviewers like your manuscript and we would like to publish a revised manuscript in Development, provided that the referees' comments can be satisfactorily addressed. Please attend to all of the reviewers' comments in your revised manuscript and detail them in your point-by-point response. If you do not agree with any of their criticisms or suggestions explain clearly why this is so. If it would be helpful, you are welcome to contact us to discuss your revision in greater detail. Please send us a point-by-point response indicating your plans for addressing the referees' comments, and we will look over this and provide further guidance.

Reviewer 1

Advance Summary and Potential Significance to Field:

The manuscript by McNamara et al. established an improved optogenetic strategy to create Nodal signaling patterns in live zebrafish embryos, which achieves precise temporospatial control of Nodal signaling activity. First, by replacing the light-sensitive domain LOV with light-sensitive heterodimerizing pair Cry2/CIB1N and sequestering the Type II receptor in the cytosol, called optoNodal2 reagents in the paper, the authors validated that optoNodal2 reagents eliminate dark activity and enhance the light response kinetics. Second, by modifying a custom ultra-widefield patterning microscope and designing embryo array mounts for simultaneous live-imaging of up to 36 embryos, the authors performed spatial patterning of Nodal signaling activity and detected target gene expression accordingly. Meanwhile, for applications of this optoNodal2 toolkit, they rescued

© 2025. Published by The Company of Biologists under the terms of the Creative Commons Attribution License (https://creativecommons.org/licenses/by/4.0/).

the developmental defects in MZoep mutants. Overall, the authors improved previously established optogenetic tools in reagents and microscope to activate Nodal signaling with precise patterns in live embryos.
This new tools may be useful in many developmental biology studies.

Comments for the Author:
Major:

1.This study used wildtype and mutant embryos. It is advised to clearly label embryo types in all figures so that readers know what embryos were used.

2.In Fig. 1C-D, and Fig. S2A, pSmad2 signal appears asymmetrically and heterogeneously distributed in both optoNodal and optoNodal2 reagents injected embryos. Was it due to asymmetric passage of injected reagents or light illumination? This phenomenon should be explained. Given that the authors mentioned in the Materials and Methods section that they co-injected reagents with mCherry mRNA, it would be better to show the distribution of mCherry protein or mRNA signal.

3.The intensity of Sytox of optoNodal2 reagents injected embryos in Fig. S1A (middle) is weaker than others, which makes it hard to judge whether pSmad2 signal in optoNodal2 reagents injected embryos is indeed weaker than LOV and comparable with uninjected embryos or the difference is caused by technological errors.

4.The abbreviation “hy” was used in many figures. However, there was no explanation for it anywhere. It’s better to define it when it appeared for the first time.

5.To better characterize rescue effects of optoNodal2 reagents in MZoep mutants, the statistical results of sox32 positive cell numbers in WT, MZoep, and MZoep+optoNodal2 embryos are necessary.

6.The potential limitations and future improvements of the optoNodal2 system should be discussed.

Minors:

1. In Fig. 1A, Smad2 and pSmad2 in light-on condition should be labeled in the illustration. Besides, the label “alpha-pSmad2” vs “pSmad2” should be consistent among different panels/figures.

2. Fig. S1C, definitions of class 1-3 phenotypes should be given in the main text.

3. Several panels are not indexed in the main text, such as Fig. 1E-F, Fig. 2A-B.

4. Some figures were not cited in order, such as Fig. S4 appeared before Fig. S3 and Fig. 4F appeared before Fig. 4E.

5. There were no scale bars in Fig. 4E and 4G.

6. Fig. 4G, the trunk and tail regions of the wildtype embryo with shh and hgg1 expression were inappropriately positioned so that the embryo looked abnormal.
It should be substituted with a better one.

7. Regarding embryo positioning, the term “regular array” was used. It should be explained what is regular position/array.

8. Fig. 4 legend, line 5, “---26 hpf..”.

<u>Reviewer 2</u>

Advance Summary and Potential Significance to Field:

© 2025. Published by The Company of Biologists under the terms of the Creative Commons Attribution License (https://creativecommons.org/licenses/by/4.0/).

McNamara et al. present a new optogenetic tool to induce Nodal signaling: optoNodal2. Combined with a custom ultra-widefield microscope and illumination system, this allows to tune Nodal signaling in defined spatial and temporal patterns. The authors demonstrate the utility of their new experimental pipeline by showing that optogenetic stimulation of Nodal signaling in specific embryonic regions can partially rescue defects of zebrafish Nodal mutants. All experiments are very well executed and presented. The new pipeline will be useful for a broad community beyond the zebrafish field, and the paper is therefore ideally suited as a Techniques and Resources Article.

Comments for the Author:

The work is of very high quality, and I only have a few minor suggestions:

1) The new optoNodal2 tool is very useful and convincing, but the current data does not conclusively show that it has an improved dynamic range and increased temporal and spatial resolution compared to optoNodal, as claimed in the manuscript. There are several confounding factors that make a direct comparison difficult:
- The injected mRNAs might have different half-lives (e.g. due to different codon optimalities) that have not been assessed.
- The translation efficiencies have not been directly assessed, and it is possible that one construct yields more protein (e.g. due to different codon usage).
- In the paper, the authors compare the constructs by injecting the same amounts even though the Cry2 domain in optoNodal2 is approximately three times longer than the LOV domain in optoNodal.
- Sometimes the signaling induced by optoNodal is stronger than for optoNodal2 (e.g. in Fig. S2B).
- Sometimes the background signals for optoNodal2 and optoNodal are similar (e.g. in the dark at 120 min in Fig. S2B).
- The statement "By contrast, signaling in the optoNodal reagents continued to accumulate for at least 90 minutes after cessation of illumination. We confirmed this observation by repeating the dynamic response measurements in an independent mutant background lacking Nodal signaling (MZoep, Fig. S2)" does not seem to be supported by the data shown in Fig. S2, where signaling does not accumulate for at least 90 min.

Given these confounding factors, I suggest to tone down the conclusions when comparing optoNodal2 to optoNodal.

2) I suggest to also tone down the word "rescued" to "partially rescued" wherever possible given the very strong defects still present in optogenetically stimulated mutants. Injection of Activin-encoding mRNA can almost fully rescue MZoep mutants (PMID 10199408). Are there regimes of uniform optoNodal2 stimulation that can also lead to a rescue?

3) The double-stained embryos in Fig. 2E,F suggest that flh is induced at a wider range than lft2. It would be useful if the authors could point out which expression domain corresponds to the illumination pattern, and speculate why the ranges might differ.

4) In Fig. 3B, 30 pg of optoNodal2 mRNA was used, which according to Fig. S1E induces strong phenotypes in the dark. If the authors have performed this experiment also in the dark, it would be very convincing to include it in the paper to rule out that non-specific activation of Nodal signaling is responsible for the induction of endoderm precursors.

5) It would be useful for the readers if the authors could state whether the fluctuating levels of optoNodal in Fig. 1F are reproducible. The Sytox signal in Fig. S1A also varies strongly. If the levels fluctuate due to batch variations, it would be good to consistently normalize the pSmad2 data to the Sytox intensities.

6) Given the submission as a Techniques and Resources Article, it would be useful to provide the MATLAB software as a resource to the community.

7) I could not find the plasmids with the given accession numbers on Addgene, but I assume they will be made available after publication. In the Materials and Methods section, the cloning details for how the myristoylation motif was removed from the Type II construct seems to be missing.

© 2025. Published by The Company of Biologists under the terms of the Creative Commons Attribution License (https://creativecommons.org/licenses/by/4.0/).

8) Rather than the complex cubic smoothing spline interpolations, I suggest simple line connections between data points for clarity of presentation.

9) Please show individual data points in bar charts such as Fig. S1B.

10) The Materials and Methods section states most of the experimental details, but it would be useful for the readers if the injected mRNA amounts, genetic backgrounds, stages and light intensities could be mentioned in all figure legends.

Reviewer 3

Advance Summary and Potential Significance to Field:

The authors have generated a significantly improved optogenetic tool system to activate Nodal signaling in the zebrafish embryo, which could be applied to other signaling systems as well. Compared to the previous light-activated LOV domain dimerization, they used a different dimerization system from Arabadopsis, as well as a Acvr2 intracellular domain that is cytosolic, rather than tethered to the cell membrane, which reduced the background activation of this system. They show vastly improved kinetics of Nodal inactivation when illumination is turned off. They also developed a system that allows patterned illumination within an embryo, which they elegantly apply to rescue of Nodal-signaling deficient mutant embryos. They show pSmad2 induction, cell internalization movements of gastrulation, and target gene expression as readouts of successful Nodal signaling induction and rescue.

Comments for the Author:

The manuscript can be strengthened in addressing the following points.

In Fig S1 mRNA amounts are compared for OptoNodal and OptoNodal2. However, mRNA activity can vary vastly from one batch of in vitro transcribed mRNA to the next, so comparing absolute amounts can be deceiving. The authors do provide assays subsequently that suggest similar activity, but it would be good to be explicit about similar activity levels in the text, so it is clear that it is reasonable to make these comparisons.

Some pSmad2 images in Fig 1E do not appear reflective of measurements shown in Fig 1F, e.g. 20 min Optonodal, and 65 min Optonodal2. 65 min in optonodal seems an outlier experiment, which makes one wonder about internal controls, variability in parameters, and how such a result could occur. Why would it decline so much at this timepoint and then rise up greatly at the next timepoint?

Why is pSmad2 not induced more widely in the embryo in Fig 1? Is illumination not even across each embryo? Is it due to varying orientations of each embryo?

Regarding pSmad2 mean intensities graphed, are nuclei lacking pSmad2 included in the mean or is it only nuclei above a background threshold level included in the mean? If the former, then the point above becomes a concern, that is, that only a part of the blastoderm cells are responding to the light. Based on the methods section, it seems that all segmented nuclei are included in the mean.

Why do the authors state “patterns with lower intensity resulted in higher frequencies of MZoeplike phenotypes (Fig. 4E, middle),”? The data in Fig 4F indicates the highest rescue of trunk somites and notochord at the lowest intensity. The authors also state this in the subsequent sentence, “Complex structures were most often rescued with narrow, low intensity rings of Nodal activation.” Yet the best rescue is at the highest intensity with a narrow ring (Fig 4E, top right embryo), as stated in the text. It seems there may be no consistent condition that is best and perhaps some variability in staging or other parameter leads to this. This should be discussed.

© 2025. Published by The Company of Biologists under the terms of the Creative Commons Attribution License (https://creativecommons.org/licenses/by/4.0/).

In Figure 4, the ‘severe dorsalization’ which is referred to as Nodal gain-of-function looks generally like a messed up embryo. Some analysis is needed to show ‘severe dorsalization’ or Nodal gain-of-function, as the morphology of the embryo shown could be the result of a lot of perturbations.

Minor:
Fig 1D, the 25 illumination intensity is misplaced at 20.

In Fig S2A, it would be good to be consistent with top row as Optonodal and bottom row as Optonodal2 as in Fig 1.

In Fig 4G, include the optogenetic parameters for the embryos shown.

First revision

Author response to reviewers' comments

The reviewers like your manuscript and we would like to publish a revised manuscript in Development, provided that the referees' comments can be satisfactorily addressed.
Please attend to all of the reviewers' comments in your revised manuscript and detail them in your point-by-point response. If you do not agree with any of their criticisms or suggestions explain clearly why this is so. If it would be helpful, you are welcome to contact us to discuss your revision in greater detail. Please send us a point-by-point response indicating your plans for addressing the referees’ comments, and we will look over this and provide further guidance.

We are grateful to the reviewers for their thoughtful and insightful comments on our manuscript. We are happy to hear that *Development* would like to publish a revised manuscript. We address all the reviewers’ comments in the updated manuscript. Point-by-point responses to each comment are below.

Reviewer 1:

The manuscript by McNamara et al. established an improved optogenetic strategy to create Nodal signaling patterns in live zebrafish embryos, which achieves precise temporospatial control of Nodal signaling activity. First, by replacing the light-sensitive domain LOV with light-sensitive heterodimerizing pair Cry2/CIB1N and sequestering the Type II receptor in the cytosol, called optoNodal2 reagents in the paper, the authors validated that optoNodal2 reagents eliminate dark activity and enhance the light response kinetics.
Second, by modifying a custom ultra-widefield patterning microscope and designing embryo array mounts for simultaneous live-imaging of up to 36 embryos, the authors performed spatial patterning of Nodal signaling activity and detected target gene expression accordingly. Meanwhile, for applications of this optoNodal2 toolkit, they rescued the developmental defects in MZoep mutants. Overall, the authors improved previously established optogenetic tools in reagents and microscope to activate Nodal signaling with precise patterns in live embryos. This new tools may be useful in many developmental biology studies.

We thank the Reviewer for the clear summary and positive assessment. We are glad to hear that the Reviewer believes that the tools presented in our manuscript may be useful in many other contexts.

Reviewer 1 Comments for the Author:
Major:

1. This study used wildtype and mutant embryos. It is advised to clearly label embryo types in all figures so that readers know what embryos were used.

© 2025. Published by The Company of Biologists under the terms of the Creative Commons Attribution License (https://creativecommons.org/licenses/by/4.0/).

We added genotype labels.

2. In Fig. 1C-D, and Fig. S2A, pSmad2 signal appears asymmetrically and heterogeneously distributed in both optoNodal and optoNodal2 reagents injected embryos. Was it due to asymmetric passage of injected reagents or light illumination? This phenomenon should be explained. Given that the authors mentioned in the Materials and Methods section that they co-injected reagents with mCherry mRNA, it would be better to show the distribution of mCherry protein or mRNA signal.

   The reviewer is correct that pSmad2 staining is heterogeneous in Figs. 1 and S2. This variability is most likely due to imperfect dispersion of mRNA following injection. Similar effects can be seen in other optogenetics studies (for example, see Fig. 3D in Rogers et al[1]) and more traditional embryological work (see Fig. 2D in Montague and Schier[2]). For the figure panels in question, we apply the generally accepted practice of injecting at the early 1-cell stage to give the mRNA maximal opportunity to disperse before cell cleavage. Although this practice is a field standard, we find that this technique is insufficient to achieve truly uniform distribution. We now note this issue in the legend of Fig. 1.

   In applications where maximally uniform expression is required—for example, in our rescue experiments of Fig. 4— we developed an approach to inject mRNA into each cell of a 4-cell stage embryo. 'Manually' dispersing mRNA in this manner improves distribution. This extra step was essential for the success of our rescue experiments. However, this approach is extremely labor intensive and slow. For that reason, we could not apply it to the experiments where many embryos had to be injected simultaneously. Consistent with practices in a comparable optogenetics study[1], we instead average pSmad2 staining intensities across multiple replicate embryos to detect trends in the data.

   mCherry mRNA was not injected in this experiment, so correlation of mCherry fluorescence with pSmad2 staining is not possible. mCherry mRNA injection was only performed for spatial patterning experiments, where the boundaries of the embryo had to be precisely identified via fluorescence imaging (e.g. in Figs. 3 and 4).

3. The intensity of Sytox of optoNodal2 reagents injected embryos in Fig. S1A (middle) is weaker than others, which makes it hard to judge whether pSmad2 signal in optoNodal2 reagents injected embryos is indeed weaker than LOV and comparable with uninjected embryos or the difference is caused by technological errors.

   We did not normalize immunofluorescence signal to DNA stain signal for two reasons:

   First, the physical natures of these staining approaches are very different: penetrance and labeling of Sytox stain may not correlate with penetrance and labeling of antibody stains. We illustrate this in Fig. R1, below. We made a scatter plot of the mean nuclear Sytox green signal vs. the mean pSmad2 staining intensity for 10 optoNodal2-injected embryos. If Sytox were an effective control for embryo-to-embryo variation in pSmad2 staining, we would observe a positive correlation between these measurements. However, we find that DNA stain and pSmad2 stain measurements are uncorrelated (R^2 = 0.03, p = 0.66, t-test). Normalizing pSmad2 staining intensity to DNA stain intensity will therefore introduce noise and not control for antibody stain variability.

   Second, in thick samples light scattering varies strongly with wavelength. The intensity of Sytox staining would therefore drop off more abruptly with depth than the Alexa647 antibody stain, even in a perfectly co-stained sample. This effect would confound the proposed normalization approach.

© 2025. Published by The Company of Biologists under the terms of the Creative Commons Attribution License (https://creativecommons.org/licenses/by/4.0/).

We agree with the Reviewer that additional analysis could provide stronger support for the claim that the original optoNodal receptors have more dark activity than the optoNodal2 receptors. To address this concern, we repeated the measurement of background activity with enhanced sampling (10 embryos per condition). We again find that the optoNodal receptors have elevated pSmad2 staining in the dark relative to optoNodal2 receptors. We updated Fig. S1 to include these data and analyses.

To address the broader concern, we emphasize that the most important indicator of problematic background activity is at the phenotype level (i.e. in Fig. S1C). At a dose of 15 pg/receptor, the optoNodal2 receptors are well- tolerated while the optoNodal receptors exhibit phenotypic defects characteristic of hyperactive Nodal signaling. We believe that this is the strongest illustration of the difference in background activity between the reagents.

Fig. R1. Sytox intensity does not explain variance in pSmad2 staining. The scatterplot shows mean nuclear pSmad2 intensity vs. mean DNA stain intensity (Sytox). Sytox staining intensity does not explain a significant portion of the variance in pSmad2 staining intensity (R^2 = 0.03).

4. The abbreviation “hy” was used in many figures. However, there was no explanation for it anywhere. It’s better to define it when it appeared for the first time.

   The abbreviation ‘hy’ is common in the physics and optogenetics literature to indicate the addition of light. We updated the manuscript to make this explicit in the first instance (i.e. in the legend of Fig. 2).

5. To better characterize rescue effects of optoNodal2 reagents in MZoep mutants, the statistical results of sox32 positive cell numbers in WT, MZoep, and MZoep+optoNodal2 embryos are necessary.

   Our intent in Fig.3 was not to claim that we rescue the full wild-type number and distribution of *sox32*+ cells in patterned MZ*oep* embryos. Indeed, Fig. 3 shows that we do not. Rather, we intended to show that optogenetic stimulation could result in sufficient signaling to induce endodermal gene expression— traditionally regarded as a ‘high-threshold’ Nodal target— and that this induced endoderm would engage in internalization movements. We believe that these claims are supported by the data in Fig. 3.

   We agree with the Reviewer that the extent of rescue could be better described. To this end, we repeated the endodermal cell rescue experiment of Fig. 3 withdeeper sampling in wild-type and MZ*oep* embryos injected with optoNodal2 receptors (both light treated and dark controls). These results, now included in Fig. S7, demonstrate (1) that the amount of endoderm induced in patterned MZ*oep* embryos is reduced relative to wild-type embryos at 8 hpf, and (2) that endoderm is not induced in optoNodal2-injected MZ*oep* embryos that were left in the dark.

6. The potential limitations and future improvements of the optoNodal2 system should be discussed.

   In the Discussion, we added a section that addresses the limitations and future avenues for improvement of our system. We focus on challenges and opportunities for spatially patterning in three dimensions and spectral multiplexing of optogenetic tools.

Minor:

© 2025. Published by The Company of Biologists under the terms of the Creative Commons Attribution License (https://creativecommons.org/licenses/by/4.0/).

1. In Fig. 1A, Smad2 and pSmad2 in light-on condition should be labeled in the illustration. Besides, the label “alpha-pSmad2” vs “pSmad2” should be consistent among different panels/figures.

Done.

2. Fig. S1C, definitions of class 1-3 phenotypes should be given in the main text.

Done.

3. Several panels are not indexed in the main text, such as Fig. 1E-F, Fig. 2A- B.

Fixed.

4. Some figures were not cited in order, such as Fig. S4 appeared before Fig. S3 and Fig. 4F appeared before Fig. 4E.

We rearranged the text so all figure panels are referred to in order.

5. There were no scale bars in Fig. 4E and 4G.

Fixed.

6. Fig. 4G, the trunk and tail regions of the wildtype embryo with shh and hgg1 expression were inappropriately positioned so that the embryo looked abnormal. It should be substituted with a better one.

These embryos were distorted during fixation, presumably due to packing in the tube. We believe that the relevant structures are clearly visible, and do not believe that repeating the staining will add clarity. We can repeat the staining with fresh embryos if the Referee believes this to be a necessary correction.

7. Regarding embryo positioning, the term “regular array” was used. It should be explained what is regular position/array.

We added a clearer description of how embryos were mounted for patterning.

8. Fig. 4 legend, line 5, “---26 hpf..”.

Fixed.

Reviewer 2:

McNamara et al. present a new optogenetic tool to induce Nodal signaling: optoNodal2. Combined with a custom ultra-widefield microscope and illumination system, this allows to tune Nodal signaling in defined spatial and temporal patterns. The authors demonstrate the utility of their new experimental pipeline by showing that optogenetic stimulation of Nodal signaling in specific embryonic regions can partially rescue defects of zebrafish Nodal mutants. All experiments are very well executed and presented. The new pipeline will be useful for a broad community beyond the zebrafish field, and the paper is therefore ideally suited as a Techniques and Resources Article.

The work is of very high quality, and I only have a few minor suggestions:

We are glad that the reviewer finds the work to be of very high quality.

1. The new optoNodal2 tool is very useful and convincing, but the current data does not conclusively show that it has an improved dynamic range and increased temporal and spatial resolution compared to optoNodal, as claimed in the manuscript. There are

© 2025. Published by The Company of Biologists under the terms of the Creative Commons Attribution License (https://creativecommons.org/licenses/by/4.0/).

several confounding factors that make a direct comparison difficult:

- The injected mRNAs might have different half-lives (e.g. due to different codon optimalities) that have not been assessed.

- The translation efficiencies have not been directly assessed, and it is possible that one construct yields more protein (e.g. due to different codon usage).

- In the paper, the authors compare the constructs by injecting the same amounts even though the Cry2 domain in optoNodal2 is approximately three times longer than the LOV domain in optoNodal.

- Sometimes the signaling induced by optoNodal is stronger than for optoNodal2 (e.g. in Fig. S2B).

We agree that each of these factors complicates comparison of the reagents. To address these critiques, we remeasured the dynamic range and dark activity of both sets of receptors under modified dosage regimes.

The Reviewer correctly notes the difficulty to dose-match different optogenetic reagents (protein lifetime may also differ). We also note that different optogenetic tools may, in principle, have different 'optimal' expression levels due to biochemical differences (e.g. in photocycle, affinity, or kinase activity). An alternative to dose-matching is to compare different tools at their respective optimal doses. We consider the optimal dose as the highest dose that does not cause adverse phenotypes.

We determined optimal dosage for each receptor by repeating the experiments in Fig. S1C over a larger dosage range. 15 pg/receptor is the maximum tolerable dose for optoNodal2, and 3.75 pg/receptor is the maximum tolerable dose for optoNodal. We then injected receptors at these optimized doses and compared (1) maximal signaling activity after 1 hour of saturating illumination and (2) background activity in the absence of light stimulation for stage-matched embryos. As predicted, we find that both receptors exhibit comparable background activity, but the optoNodal2 receptors exhibit higher maximal signaling activity - consistent with a greater dynamic range for the optoNodal2 system.

This comparison does not match the reagent doses 'molecule for molecule', but we believe it is the fairest comparison. At the optimal dosing, optoNodal2 has better dynamic range than optoNodal. These results have been added to Fig. S2 in the revised manuscript and are now referenced in the main text.

- Sometimes the background signals for optoNodal2 and optoNodal are similar (e.g. in the dark at 120 min in Fig. S2B).

The reviewer is correct that the data in Fig. S2B do not show a marked difference in dark activity between the reagents. However, in aggregate our data show more dark activity in optoNodal than in optoNodal2. This conclusion is supported by independent experiments of pSmad2 levels in Fig. 1D and Fig. 1F, as well as phenotypic data in Fig. S1C. We also added an independent, more deeply sampled comparison of Nodal signaling activity in the dark as part of a response to a comment from Reviewer 1. This experiment also shows that optoNodal dark activity exceeds that of optoNodal2, when injected at an equal mRNA doses. This improved analysis is now presented in Figs. S1 A and B.

- The statement "By contrast, signaling in the optoNodal reagents continued to accumulate for at least 90 minutes after cessation of illumination. We confirmed this observation by repeating the dynamic response measurements in an independent mutant background lacking Nodal signaling (MZoep, Fig. S2)" does not seem to be

© 2025. Published by The Company of Biologists under the terms of the Creative Commons Attribution License (https://creativecommons.org/licenses/by/4.0/).

supported by the data shown in Fig. S2, where signaling does not accumulate for at least 90 min.

The Reviewer is correct. We changed "continues to accumulate" to "fails to return to background levels" to more accurately describe our observations.

Given these confounding factors, I suggest to tone down the conclusions when comparing optoNodal2 to optoNodal.

We added more comparison data to the manuscript and we revised the text to include a more nuanced and specific discussion of the relative merits of the reagents.

2) I suggest to also tone down the word "rescued" to "partially rescued" wherever possible given the very strong defects still present in optogenetically stimulated mutants. Injection of Activin-encoding mRNA can almost fully rescue MZoep mutants (PMID 10199408). Are there regimes of uniform optoNodal2 stimulation that can also lead to a rescue?

We adjusted the language to indicate that rescue was partial and we now highlight remaining phenotypic deficits.

We have tried uniform illumination of optoNodal2-injected MZ*oep* embryos under varying illumination doses and schedules. We failed to create rescue at any condition tested. Instead, phenotypic deficits characteristic of Nodal gain-of-function are common. We are aware of the *activin* injection results the Reviewer references. We suspect that this result could have arisen from the method of injection. In the paper's methods section, it was stated that mRNAs were '*microinjected into the yolk or blastomeres at 1-4 cell stage*'. Injection of mRNA into the vegetal yolk at the 4-cell stage can lead to significant enrichment in the YSL, a physiological source of Nodal ligands. It is therefore possible that injection in this manner leads to a near normal distribution of Nodal activity due to localized secretion of Activin from the YSL.

3) The double-stained embryos in Fig. 2E,F suggest that flh is induced at a wider range than lft2. It would be useful if the authors could point out which expression domain corresponds to the illumination pattern, and speculate why the ranges might differ.

The Reviewer is correct that *flh* is induced over a wider spatial range than *lft2*. We hypothesize that one source of this effect is differential sensitivity to Nodal signaling. Measurements from the Schier lab[3] demonstrate that *flh* is induced at lower Nodal concentrations than *lft2*. In our projection system, optical aberrations and light scattering cause some light to leak beyond the nominal edges of the projection patterns. We suspect that *flh* responds more sensitively to this off-target light than does *lft2*.

To test this interpretation, we repeated our patterning experiment with an imposed gradient of light. These experiments demonstrated that light-induced Nodal signaling causes a more sensitive response in *flh* than in *lft2* (see new Supplemental Fig. S6 and description in the main text).

4) In Fig. 3B, 30 pg of optoNodal2 mRNA was used, which according to Fig. S1E induces strong phenotypes in the dark. If the authors have performed this experiment also in the dark, it would be very convincing to include it in the paper to rule out that non-specific activation of Nodal signaling is responsible for the induction of endoderm precursors.

Good point. We added a supplementary figure with quantification of a replicate endoderm patterning experiment (Fig. S7). We include a panel demonstrating that unilluminated MZ*oep* embryos injected with 30 pg of optoNodal2 receptor mRNA do not specify endodermal cells.

© 2025. Published by The Company of Biologists under the terms of the Creative Commons Attribution License (https://creativecommons.org/licenses/by/4.0/).

5) It would be useful for the readers if the authors could state whether the fluctuating levels of optoNodal in Fig. 1F are reproducible. The Sytox signal in Fig. S1A also varies strongly. If the levels fluctuate due to batch variations, it would be good to consistently normalize the pSmad2 data to the Sytox intensities.

A similar concern was raised by Reviewer 1. We copy our response to that comment here:

> **We did not normalize immunofluorescence signal to DNA stain signal for two reasons:**
>
> **First, the physical natures of these staining approaches are very different: penetrance and labeling of Sytox stain may not correlate with penetrance and labeling of antibody stains. We illustrate this in Fig. R1, below. We made a scatter plot of the mean nuclear Sytox green signal vs. the mean pSmad2 staining intensity for 10 optoNodal2-injected embryos. If Sytox were an effective control for embryo-to-embryo variation in pSmad2 staining, we would observe a positive correlation between these measurements. However, we find that DNA stain and pSmad2 stain measurements are uncorrelated (R^2 = 0.03, p = 0.66, t-test). Normalizing pSmad2 staining intensity to DNA stain intensity will therefore introduce noise and not control for antibody stain variability.**
>
> **Second, in thick samples light scattering varies strongly with wavelength. The intensity of Sytox staining would therefore drop off more abruptly with depth than the Alexa647 antibody stain, even in a perfectly co-stained sample. This effect would confound the proposed normalization approach.**
>
> **We agree with the Reviewer that additional analysis could provide stronger support for the claim that the original optoNodal receptors have more dark activity than the optoNodal2 receptors. To address this concern, we repeated the measurement of background activity with enhanced sampling (10 embryos per condition). We again find that the optoNodal receptors have elevated pSmad2 staining in the dark relative to optoNodal2 receptors. We updated Fig. S1 to include these data and analyses.**
>
> **To address the broader concern, we emphasize that the most important indicator of problematic background activity is at the phenotype level (i.e. in Fig. S1C). At a dose of 15 pg/receptor, the optoNodal2 receptors are well-tolerated while the optoNodal receptors exhibit phenotypic defects characteristic of hyperactive Nodal signaling. We believe that this is the strongest illustration of the difference in background activity between the reagents.**

© 2025. Published by The Company of Biologists under the terms of the Creative Commons Attribution License (https://creativecommons.org/licenses/by/4.0/).

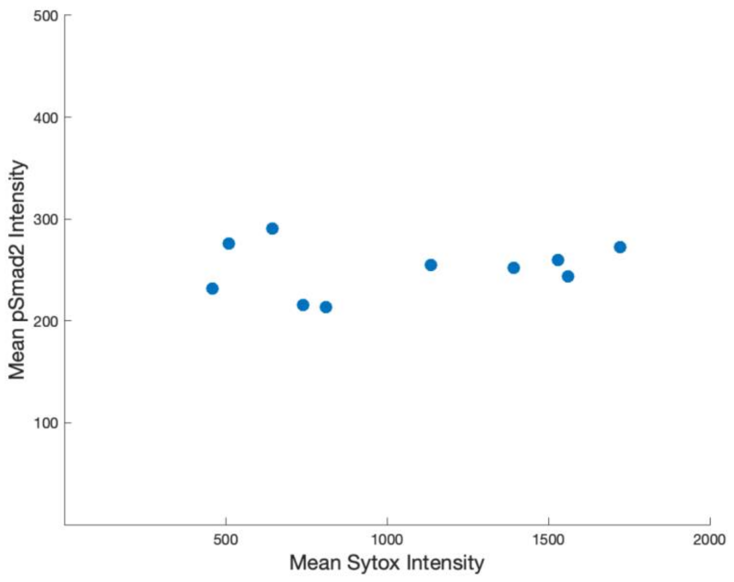

<u>Fig. R1. Sytox intensity does not explain variance in pSmad2 staining.</u> The scatterplot shows mean nuclear pSmad2 intensity vs. mean DNA stain intensity (Sytox). Sytox staining intensity does not explain a significant portion of the variance in pSmad2 staining intensity (R^2 = 0.03).

6) Given the submission as a Techniques and Resources Article, it would be useful to provide the MATLAB software as a resource to the community.

We will distribute our control and GUI code on the Lord Lab's GitHub site upon publication. The only omitted code will be in situations where agreements with hardware vendors preclude providing access to proprietary developer tools.

7) I could not find the plasmids with the given accession numbers on Addgene, but I assume they will be made available after publication. In the Materials and Methods section, the cloning details for how the myristoylation motif was removed from the Type II construct seems to be missing.

All plasmids are now available on Addgene.

Cloning details on how myristoylation motif was removed from the Type II construct have now been added to the Materials and Methods section.

8) Rather than the complex cubic smoothing spline interpolations, I suggest simple line connections between data points for clarity of presentation.

We believe that a smoothing interpolation provides a more accurate representation of the data. The linear interpolations are conceptually simpler, but over-emphasize small fluctuations resulting from finite sampling. We do not believe that this choice alters any of the conclusions of the paper, and therefore prefer to keep this aspect of the data presentation as is.

9) Please show individual data points in bar charts such as Fig. S1B.

Individual data points have been added to the bar chart of Fig. S1B.

10) The Materials and Methods section states most of the experimental details, but it would be useful for the readers if the injected mRNA amounts, genetic backgrounds, stages and light intensities could be mentioned in all figure legends.

© 2025. Published by The Company of Biologists under the terms of the Creative Commons Attribution License (https://creativecommons.org/licenses/by/4.0/).

We added mRNA injection dosages, genetic backgrounds, embryo stages and light intensities from optogenetic treatments to each relevant figure legend.

Reviewer 3:

The authors have generated a significantly improved optogenetic tool system to activate Nodal signaling in the zebrafish embryo, which could be applied to other signaling systems as well. Compared to the previous light-activated LOV domain dimerization, they used a different dimerization system from Arabadopsis, as well as a Acvr2 intracellular domain that is cytosolic, rather than tethered to the cell membrane, which reduced the background activation of this system. They show vastly improved kinetics of Nodal inactivation when illumination is turned off. They also developed a system that allows patterned illumination within an embryo, which they elegantly apply to rescue of Nodal-signaling deficient mutant embryos. They show pSmad2 induction, cell internalization movements of gastrulation, and target gene expression as readouts of successful Nodal signaling induction and rescue.

We are grateful that the reviewer finds our optogenetic tool system to be 'significantly improved' compared to previously available tools.

In Fig S1 mRNA amounts are compared for OptoNodal and OptoNodal2. However, mRNA activity can vary vastly from one batch of in vitro transcribed mRNA to the next, so comparing absolute amounts can be deceiving. The authors do provide assays subsequently that suggest similar activity, but it would be good to be explicit about similar activity levels in the text, so it is clear that it is reasonable to make these comparisons.

The reviewer is correct regarding the difficulties of comparing two different *in vitro* transcribed reagents. Reviewer 2 also raised a similar issue. We copy the response to to Reviewer 2, comment 1 below.

We agree that each of these factors complicates comparison of the reagents. To address these critiques, we remeasured the dynamic range and dark activity of both sets of receptors under modified dosage regimes.

The Reviewer correctly notes the difficulty to dose-match different optogenetic reagents (protein lifetime may also differ). We also note that different optogenetic tools may, in principle, have different 'optimal' expression levels due to biochemical differences (e.g. in photocycle, affinity, or kinase activity). An alternative to dose-matching is to compare different tools at their respective optimal doses. We consider the optimal dose as the highest dose that does not cause adverse phenotypes.

We determined optimal dosage for each receptor by repeating the experiments in Fig. S1C over a larger dosage range. 15 pg/receptor is the maximum tolerable dose for optoNodal2, and 3.75 pg/receptor is the maximum tolerable dose for optoNodal. We then injected receptors at these optimized doses and compared (1) maximal signaling activity after 1 hour of saturating illumination and (2) background activity in the absence of light stimulation for stage-matched embryos. As predicted, we find that both receptors exhibit comparable background activity, but the optoNodal2 receptors exhibit higher maximal signaling activity - consistent with a greater dynamic range for the optoNodal2 system.

This comparison does not match the reagent doses 'molecule for molecule', but we believe it is the fairest comparison. At the optimal dosing, optoNodal2 has better dynamic range than optoNodal. These results have been added to Fig. S2 in the revised manuscript and are now referenced in the main text.

Some pSmad2 images in Fig 1E do not appear reflective of measurements shown in Fig 1F, e.g. 20 min Optonodal, and 65 min Optonodal2. 65 min in optonodal seems an outlier experiment, which makes one wonder about internal controls, variability in parameters, and how such a

© 2025. Published by The Company of Biologists under the terms of the Creative Commons Attribution License (https://creativecommons.org/licenses/by/4.0/).

result could occur. Why would it decline so much at this timepoint and then rise up greatly at the next timepoint?

For the panels in Fig. 1C and E we selected the embryos with the median average nuclear pSmad2 staining intensity. We believe that this practice was more fair than manually selecting embryos that best 'illustrate' the quantifications. The datapoints in Figs. 1D and F depict the *average* of replicate embryos; the median and average can differ.

We suspect that the fluctuation at 65 min is just noise. Embryo-to-embryo variability was driven by variability in the mRNA dose delivered via microinjection. Further, the mRNA does not perfectly distribute through the embryo even when injected at the early 1-cell stage (see response to Reviewer 1, comment 2). We performed as many replicates as was feasible. Each experiment in Fig. 1 involved gathering full confocal stacks of ~60 embryos across conditions. Each datapoint comprises an average of 5 embryos, and so there are statistical fluctuations.

We replicated this experiment in an independent Nodal loss-of-function background in Fig. S3. This replicate supports the conclusions drawn from the experiments in Fig. 1 E and F.

Why is pSmad2 not induced more widely in the embryo in Fig 1? Is illumination not even across each embryo? Is it due to varying orientations of each embryo?

The reviewer is justified in pointing out the heterogeneity in pSmad2 staining in the embryos of Fig. 1; Reviewer 1 also raised this point. The source of this variability is most likely due to imperfect dispersion of mRNA following injection. Similar effects can be seen in other optogenetics studies (for example, see Fig. 3D in Rogers et al[1]) and more traditional embryological work (see Fig. 2D in Montague and Schier[2]). For the figure panels in question, we apply the generally accepted practice of injecting at the early 1-cell stage to give the mRNA maximal opportunity to disperse before cell cleavage. However, we find that this practice is insufficient to achieve truly uniform distribution. We now make note of this issue in the legend of Fig. 1.

Regarding pSmad2 mean intensities graphed, are nuclei lacking pSmad2 included in the mean or is it only nuclei above a background threshold level included in the mean? If the former, then the point above becomes a concern, that is, that only a part of the blastoderm cells are responding to the light. Based on the methods section, it seems that all segmented nuclei are included in the mean.

We plotted the mean of *all* segmented nuclei in the embryos. This approach avoids a poorly constrained choice of threshold to define pSmad2-positive cells. This same averaging approach was applied in a comparable recent study presenting new optogenetic reagents in zebrafish[1].

Why do the authors state "patterns with lower intensity resulted in higher frequencies of MZoep like phenotypes (Fig. 4E, middle),"? The data in Fig 4F indicates the highest rescue of trunk somites and notochord at the lowest intensity. The authors also state this in the subsequent sentence, "Complex structures were most often rescued with narrow, low intensity rings of Nodal activation." Yet the best rescue is at the highest intensity with a narrow ring (Fig 4E, top right embryo), as stated in the text. It seems there may be no consistent condition that is best and perhaps some variability in staging or other parameter leads to this. This should be discussed.

The Reviewer is correct that optogenetic rescue is variable. We address the issues raised by the Reviewer below.

Yes, somites and notochord were most often rescued with low intensity optogenetic treatment. However, embryos with MZ*oep*-like phenotypes also occurred in these

© 2025. Published by The Company of Biologists under the terms of the Creative Commons Attribution License (https://creativecommons.org/licenses/by/4.0/).

treatments, while MZ*oep*-like phenotypes were absent in the high-intensity treatments. In the high-intensity conditions, 'failed' rescues instead showed severe dorsalization. When we stated that "*patterns with lower intensity resulted in higher frequencies of MZoep like phenotypes*", we were referring to the fact that this outcome was only observed in low intensity conditions. To clarify the distinction between different failed rescues, we added a 'Nodal Loss of Function' phenotype frequency table to Fig. 4F.

The Reviewer is correct that the single best rescue occurred at high intensity with a narrow ring. However, complex structures—somites and notochord— appeared with highest frequency with thin, low-intensity rings. The trends in the data that we report were consistent across replicates of the optogenetic rescue.

In the revised submission we discuss the variability in the rescue. In the Discussion we now discuss the sources of variability in the optogenetic rescues and our plans to improve them.

In Figure 4, the 'severe dorsalization' which is referred to as Nodal gain-of-function looks generally like a messed up embryo. Some analysis is needed to show 'severe dorsalization' or Nodal gain-of-function, as the morphology of the embryo shown could be the result of a lot of perturbations.

We agree that a claim of 'dorsalization' should be supported more strongly. We adjusted the label in Fig. 4 F to read 'Hyperactive Nodal Signaling' instead. We believe that attributing these 'messed up embryo' phenotypes to hyperactive Nodal signaling is warranted. Our negative control embryos—injected with optoNodal2 and mounted on the patterning microscope, but not illuminated— differ from the treatment embryos *only* in being illuminated, and still exhibit MZ*oep*-like phenotypes with 100% frequency. All other perturbations that could lead to phenotypic problems (e.g. injection, stresses associated with embryo mounting or retrieval, dark activity) are shared between the treatment and control embryos.

Fig 1D, the 25 illumination intensity is misplaced at 20.

The plot has been corrected.

In Fig S2A, it would be good to be consistent with top row as Optonodal and bottom row as Optonodal2 as in Fig 1.

Done.

In Fig 4G, include the optogenetic parameters for the embryos shown.

Done.

<u>References:</u>

1. Rogers, K. W., ElGamacy, M., Jordan, B. M. & Müller, P. Optogenetic investigation of BMP target gene expression diversity. *eLife* **9**, e58641 (2020).

2. Montague, T. G. & Schier, A. F. Vg1-Nodal heterodimers are the endogenous inducers of mesendoderm. *Elife* **6**, e28183 (2017).

3. Dubrulle, J. *et al.* Response to Nodal morphogen gradient is determined by the kinetics of target gene induction. *eLife* **4**, e05042 (2015).

© 2025. Published by The Company of Biologists under the terms of the Creative Commons Attribution License (https://creativecommons.org/licenses/by/4.0/).

Second decision letter

MS ID#: dev.204506R1

MS TITLE: Optogenetic control of Nodal signaling patterns

AUTHORS: Harold M. McNamara; Alison M. Guyer; Bill Z. Jia; Vicente J. Parot; Caleb D. Dobbs; Alexander F. Schier; Adam E. Cohen; Nathan D. Lord

Apologies for the delay in obtaining reviews on your revised paper. Your will be pleased to see that there are just a couple of minor comments/suggestions for you to consider before we proceed to publication. The referees' comments are appended below, or you can access them online: please go to .

Reviewer 1

The authors have appropriately addressed all reviewers' comments. Excellent paper!

Reviewer 2

Advance summary and potential significance to field

The authors have generated a significantly improved optogenetic tool system to activate Nodal signaling in the zebrafish embryo, which could be applied to other signaling systems as well. Compared to the previous light-activated LOV domain dimerization, they used a different dimerization system from Arabadopsis, as well as a Acvr2 intracellular domain that is cytosolic, rather than tethered to the cell membrane, which reduced the background activation of this system. They show vastly improved kinetics of Nodal inactivation when illumination is turned off. They also developed a system that allows patterned illumination within an embryo, which they elegantly apply to rescue of Nodal-signaling deficient mutant embryos. They show pSmad2 induction, cell internalization movements of gastrulation, and target gene expression as readouts of successful Nodal signaling induction and rescue.

Comments for the author

Fig 1C-F. By averaging all the negative pSmad2 nuclei that likely did not receive the mRNA due to dispersal variations, the authors potentially distort their results. The n's are quite low, so if by chance one or more timepoints has less well dispersal or very broad dispersal, it will be distorted to a lower or higher value, respectively. The repetition of this experiment in Fig S3 shows some differences in kinetics for the Optonodal signal decline, which may reflect this issue (or another). A response that other's have done it this way, does not make it a good or the most rigorous method, which should be strived for. Others have measured the nuclei average above a background threshold in such types of experiments to avoid this issue. The authors' stated point is to examine kinetics and intensity responses in these assays, so including negative nuclei that likely did not receive the mRNA does not seem appropriate, even if others have done it in the past. The authors could simply reanalyze their data, it requires no additional experiment, or provide a rationale in the text or methods for including the negative nuclei considering the mRNA dispersal issue.

In Fig 1C and E, one would expect the Sytox staining to be equivalent between all embryos shown, however, it is not. Just wondering what this variability may be about and if it may be affecting the other pSmad2 channel too.

Second revision

Author response to reviewers' comments

© 2025. Published by The Company of Biologists under the terms of the Creative Commons Attribution License (https://creativecommons.org/licenses/by/4.0/).

Reviewer 1: The authors have appropriately addressed all reviewers' comments. Excellent paper!

We thank the reviewer for their time and the favorable review of our work.

Reviewer 2: SUMMARY OF THE ADVANCE MADE IN THIS PAPER AND ITS POTENTIAL SIGNIFICANCE TO THE FIELD

The authors have generated a significantly improved optogenetic tool system to activate Nodal signaling in the zebrafish embryo, which could be applied to other signaling systems as well. Compared to the previous light-activated LOV domain dimerization, they used a different dimerization system from Arabadopsis, as well as a Acvr2 intracellular domain that is cytosolic, rather than tethered to the cell membrane, which reduced the background activation of this system. They show vastly improved kinetics of Nodal inactivation when illumination is turned off. They also developed a system that allows patterned illumination within an embryo, which they elegantly apply to rescue of Nodal-signaling deficient mutant embryos. They show pSmad2 induction, cell internalization movements of gastrulation, and target gene expression as readouts of successful Nodal signaling induction and rescue.

We thank the reviewer for their continued input and effort. We address the two specific comments below.

SUGGESTIONS TO AUTHORS

Fig 1C-F. By averaging all the negative pSmad2 nuclei that likely did not receive the mRNA due to dispersal variations, the authors potentially distort their results. The n's are quite low, so if by chance one or more timepoints has less well dispersal or very broad dispersal, it will be distorted to a lower or higher value, respectively. The repetition of this experiment in Fig S3 shows some differences in kinetics for the Optonodal signal decline, which may reflect this issue (or another). A response that other's have done it this way, does not make it a good or the most rigorous method, which should be strived for. Others have measured the nuclei average above a background threshold in such types of experiments to avoid this issue. The authors' stated point is to examine kinetics and intensity responses in these assays, so including negative nuclei that likely did not receive the mRNA does not seem appropriate, even if others have done it in the past. The authors could simply reanalyze their data, it requires no additional experiment, or provide a rationale in the text or methods for including the negative nuclei considering the mRNA dispersal issue.

Per the reviewer's suggestion, we have repeated the analysis of Fig. 1 panels D and F, this time only including nuclei with a–pSmad2 staining intensity above a threshold. Thresholds were chosen based on uninjected (panel D intensity series) or unilluminated (panel F time series t = 0 condition) control embryos; only nuclei brighter than 90% of the negative control nuclei were selected for averaging.

The modified versions of these figure panels are below. This procedure does not alter the key conclusions of either panel. Some numerical values are modestly changed, but the overall trends referenced in the main text remain the same. The only exception is the 20-minute timepoint for optoNodal reagents in the time series data (panel B below). This datapoint shifted up noticeably, resulting in a small alteration in the cubic spline interpolation. We believe that this likely reflects increased influence of an outlier embryo caused by the reduction in dataset size due to thresholding. However, this change does not alter the key conclusion of the experiment, which is that optoNodal reagents have slower shut-off kinetics than our optoNodal2 reagents.

We appreciate the reviewer's suggestion for additional analysis that strengthens our conclusions. However, we believe that our prior analysis approach is preferable. Whatever errors may have been introduced from imperfect RNA distribution were uncorrelated with the choice of construct we injected. Hence, these errors did not introduce a systematic bias. Thresholding based on the quantity we are trying to measure, however, undercounts the cells with the lowest expression level. For this reason, we prefer to keep Fig. 1 as presented.

As suggested by the reviewer, we now provide a rationale for our approach in the methods. In the

© 2025. Published by The Company of Biologists under the terms of the Creative Commons Attribution License (https://creativecommons.org/licenses/by/4.0/).

methods section *'Fixed embryo staining, imaging and quantification'*, we now write: *'As noted in the legend of Fig. 1, we observe variation in illumination responses within individual embryos. This variability likely represents imperfect dispersal of optoNodal or optoNodal2 mRNA after microinjection. We elected to include all segmented nuclei in the presented quantifications, rather than attempt to exclude nuclei from cells with putatively low mRNA dosages. We believe this to be the most rigorous practice for two reasons. First, mRNA distribution is uncorrelated with the choice of construct being injected. These errors therefore did not introduce a systematic bias. Second, thresholding based on the quantity we are attempting to measure (i.e. pSmad2 staining intensity) undercounts cells with low expression levels.'*

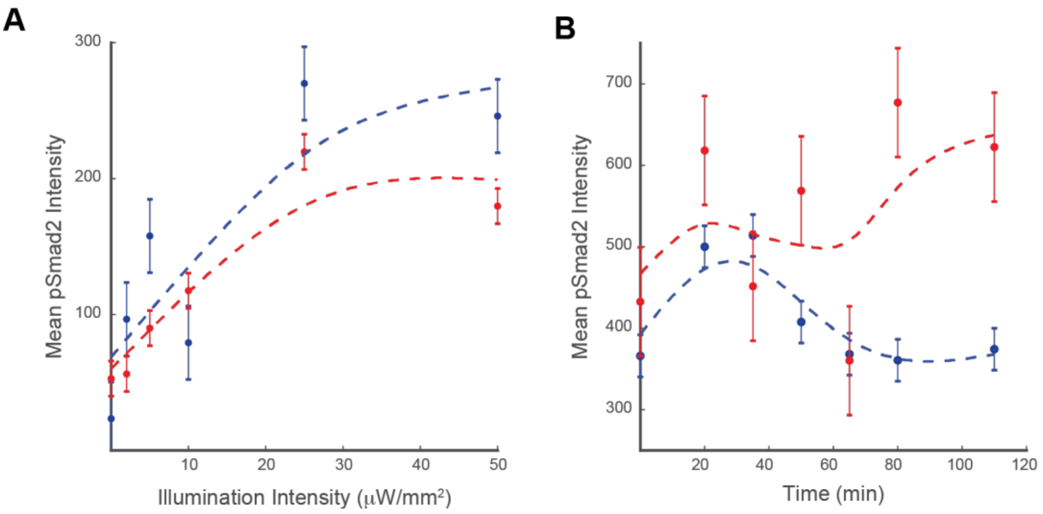

<u>Fig. 1. Reanalysis of optogenetic reagent illumination responses</u>. (A) Blue light intensity responses for optoNodal (red) and optoNodal2 (blue) reagents. a-pSmad2 staining intensities were extracted from segmented nuclei with intensities above a pre-defined threshold. Each point represents the average nuclear staining intensity from replicate embryos. Error bars denote the standard error of the mean. Dashed curves depict cubic smoothing spline interpolations. (B) Measurement of response kinetics for optoNodal (red) and optoNodal2 (blue) reagents. a- pSmad2 staining intensities were extracted from segmented nuclei with intensities above a pre-defined threshold. Each point represents the average nuclear staining intensity from replicate embryos Error bars denote the standard error of the mean. Dashed curves depict cubic smoothing spline interpolations.

In Fig 1C and E, one would expect the Sytox staining to be equivalent between all embryos shown, however, it is not. Just wondering what this variability may be about and if it may be affecting the other pSmad2 channel too.

We have noted some variability between whole-mount embryos when stained with Sytox green in this study and our previous work. We suspect that it relates to extended embryo storage times prior to fluorescence imaging. Each of the referenced experiments requires imaging ~50 large Z-stacks over multiple channels on a laser scanning confocal microscope. On our instruments, this can take up to 5 hours. Some samples are imaged immediately, but others are imaged hours later, creating an opportunity for Sytox to leach out. In our hands, DAPI exhibits less variability (see e.g. Lord et al eLife 2021). However, we selected Sytox green for this application because whole-mount embryos require large Z-stacks. The scattering associated with the short excitation and emission wavelengths of DAPI make it ill-suited for this purpose. Because none of our analysis relies on the quantitative features of Sytox staining, we do not believe that this phenomenon limits our analysis.

We do not believe that the variation in Sytox staining intensity affects the pSmad2 staining. If Sytox were affecting pSmad2— or even correlating with a hidden factor that also affects pSmad2 staining— we would expect to see a correlation in these quantities across replicate embryos. To test this idea, we scattered the mean nuclear Sytox green signal to the mean pSmad2 staining intensity for 10 optoNodal2-injected embryos (Fig. R2, below). We find that DNA stain and pSmad2 stain measurements are uncorrelated (R^2 = 0.03, p = 0.66). For this reason, we do not favor the

© 2025. Published by The Company of Biologists under the terms of the Creative Commons Attribution License (https://creativecommons.org/licenses/by/4.0/).

practice of normalizing each nucleus's pSmad2 level to Sytox intensity. To the contrary, we believe the primary eaect of this practice would be to randomize our pSmad2 measures, clouding useful comparisons.

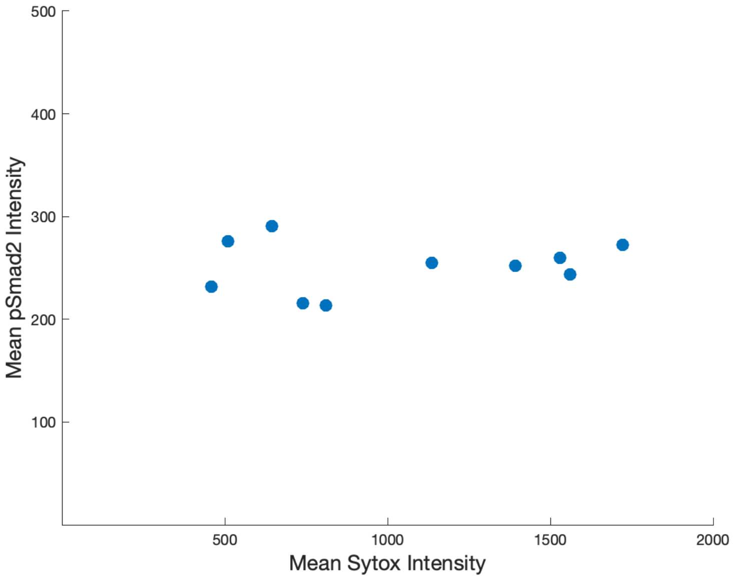

Fig. R2. Sytox intensity does not explain variance in pSmad2 staining. The above plot scatters mean nuclear pSmad2 intensity against mean DNA stain intensity (Sytox). Sytox staining intensity does not explain a significant portion of the variance in pSmad2 staining intensity (R^2 = 0.03).

Third decision letter

MS ID#: dev.204506R2

MS TITLE: Optogenetic control of Nodal signaling patterns

AUTHORS: Harold M. McNamara; Alison M. Guyer; Bill Z. Jia; Vicente J. Parot; Caleb D. Dobbs; Alexander F. Schier; Adam E. Cohen; Nathan D. Lord
ARTICLE TYPE: Research Article

I am happy to tell you that your manuscript has been accepted for publication in Development, pending our standard publication integrity checks.

© 2025. Published by The Company of Biologists under the terms of the Creative Commons Attribution License (https://creativecommons.org/licenses/by/4.0/).
